# Differential Geometric Analysis of Alterations in MH α-Helices

**DOI:** 10.1002/jcc.23328

**Published:** 2013-05-24

**Authors:** Birgit Hischenhuber, Hans Havlicek, Jelena Todoric, Sonja Höllrigl-Binder, Wolfgang Schreiner, Bernhard Knapp

**Affiliations:** [a] Center for Medical Statistics, Informatics, and Intelligent Systems, Section for Biosimulation and Bioinformatics, Medical University of ViennaVienna, Austria; [b] Faculty of Mathematics and Geoinformation, Institute of Discrete Mathematics and Geometry, Research Group for Differential Geometry and Geometric Structures, Vienna University of TechnologyVienna, Austria; [c] Laboratory of Gene Regulation and Signal Transduction, Departments of Pharmacology and Pathology, School of Medicine, University of CaliforniaCalifornia, San Diego; [d] Department of Laboratory Medicine, Medical University ViennaVienna, Austria; [e] Faculty of Mathematics and Geoinformation, Institute of Analysis and Scientific Computing, Research Group for Mathematical Modelling and Simulation, Vienna University of TechnologyVienna, Austria; [f] Department of Statistics, Protein Informatics Group, University of OxfordOxford, United Kingdom E-mail: bernhard.knapp@stats.ox.ac.uk

**Keywords:** characterization of structural alterations, differential geometric parameters, major histocompatibility com- plex, alpha-helices, immunoinformatics

## Abstract

Antigen presenting cells present processed peptides via their major histocompatibility (MH) complex to the T cell receptors (TRs) of T cells. If a peptide is immunogenic, a signaling cascade can be triggered within the T cell. However, the binding of different peptides and/or different TRs to MH is also known to influence the spatial arrangement of the MH α-helices which could itself be an additional level of T cell regulation. In this study, we introduce a new methodology based on differential geometric parameters to describe MH deformations in a detailed and comparable way. For this purpose, we represent MH α-helices by curves. On the basis of these curves, we calculate in a first step the curvature and torsion to describe each α-helix independently. In a second step, we calculate the distribution parameter and the conical curvature of the ruled surface to describe the relative orientation of the two α-helices. On the basis of four different test sets, we show how these differential geometric parameters can be used to describe changes in the spatial arrangement of the MH α-helices for different biological challenges. In the first test set, we illustrate on the basis of all available crystal structures for (TR)/pMH complexes how the binding of TRs influences the MH helices. In the second test set, we show a cross evaluation of different MH alleles with the same peptide and the same MH allele with different peptides. In the third test set, we present the spatial effects of different TRs on the same peptide/MH complex. In the fourth test set, we illustrate how a severe conformational change in an α-helix can be described quantitatively. Taken together, we provide a novel structural methodology to numerically describe subtle and severe alterations in MH α-helices for a broad range of applications. © 2013 Wiley Periodicals, Inc.

## Introduction

In adaptive immunology T cells, especially T cell receptors (TR) play an essential role in the interaction process with antigen presenting cells (APCs). TRs are localized on the surface of T cells which can be activated upon contact with APCs. These APCs present peptide antigens via the major histocompatibility (MH) complex on their surface.^1^ The recognition of the peptide/MH (pMH) complex by the TR is essential for T cell triggering.

In this context, the interface region between the complementary determining regions (CDRs) of the TR and the α-helices which span the MH binding groove (G-domain) in combination with the presented peptide is of special interest.^2^ So far sequence-based methods have been used to investigate MH α-helices, for example, the IMGT/Collier de-Perles tool.^3^ However, it is known that even sequence identical MH α-helices can have significant structural differences.^4^ This was shown experimentally on the basis of H-2K(bm8) in complex with pBM1 and pBM8 peptides^5^ and *in silico* on the basis of I-Au in complex with altered peptide ligands from myelin basic protein.^6^

The structural basis how a single TR signaling cascade is activated remains still an unsolved question. Several different models for this process were proposed^7^ and in most of them at least subtle structural deformations of the TR/pMH interface are expected. Thus, the appropriate structural description of this interface is a crucial challenge.

To characterize such deformations, several generic protein characterization methods are available from the literature: They include solvent accessible surface area, the number and position of hydrogen bonds and interaction energies, radius of gyration, bond-angle combinations, and secondary structure assignment. Also, structural alphabets based on the bond and torsion angle of four-residue long protein fragments are available.^8^ Via combination of this alphabet and principal component analysis, the motions of proteins have been described.^9^ However, structural methods specific for MH α-helix characterization are sparse and most of the time standard methods are used to describe MH α-helices in the stationary^10^ and dynamic case.^11^,^12^ Hence, in this study we propose novel methods originating from differential geometry to investigate the spatial orientation of MH α-helices based on curve models previously published by our group.^13^ Such differential geometric methods have been applied before for several aspects of structural bioinformatics: Goldman and Wipke^14^ described the molecular surface complementarity in ligand docking. Marathe et al.^15^ used the radius of curvature and the torsion angle to compare free DNA complexes against protein-bound DNA. Shazman et al.^16^ investigated the geometry and shape of the binding interfaces of DNA and RNA complexes. Schmidt et al.^17^ investigated the relation between Gaussian curvature of membranes and bactericidal activity via membrane destabilization. Hausrath and Goriely^18^ used curvature profiles to construct atomically detailed protein models. The calculations of the curvature and torsion relating to characterize a curve is a common method: Lewiner et al.^19^ presented a method to estimate the curvature and torsion from sampled curves. However, the application of differential geometric parameters for the description of MH α-helices is still lacking.

In the current study, we show how such differential geometric parameters can be used to describe the α-helices of both MH class 1 (MH1) and MH class 2 (MH2). We present methods to describe the MH α-helices independently as well as in their relative arrangement. Subsequently, we show how our methodology sheds light on several aspects of TR/pMH interaction: First, on the geometric differences between single MH complexes and MH complexes binding a TR; second, on different MH alleles with the same peptide and the same MH alleles with different peptides; third, on spatial deformation in the same pMH by binding two different TRs; and fourth, on helical disruption arising during a Molecular Dynamics (MD) simulation.

## Methods

### Differential geometric parameters for MH α-helices

We have shown in a previous study,^13^ how MH α-helices can be fitted by polynomials and curves in an appropriate way by application of the corrected Akaike-criterion.^20^ In the following, we present several different differential geometric methods of how these curves can be described and compared to each other. The following methods are implemented according to the mathematical background provided by Pottmann and Wallner,^21^ which is a basic introduction to differential geometry. For further details also see Do Carmo,^22^ who introduced the differential geometry especially in the Euclidean three-dimensional space.

### Curvature and torsion of α-helices

The first parameter is the local *curvature*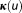, which we determined for each curve representing an α-helix. For a given parameterized curve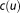, the local curvature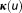 is defined as


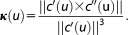
(1)

**Figure 1:**
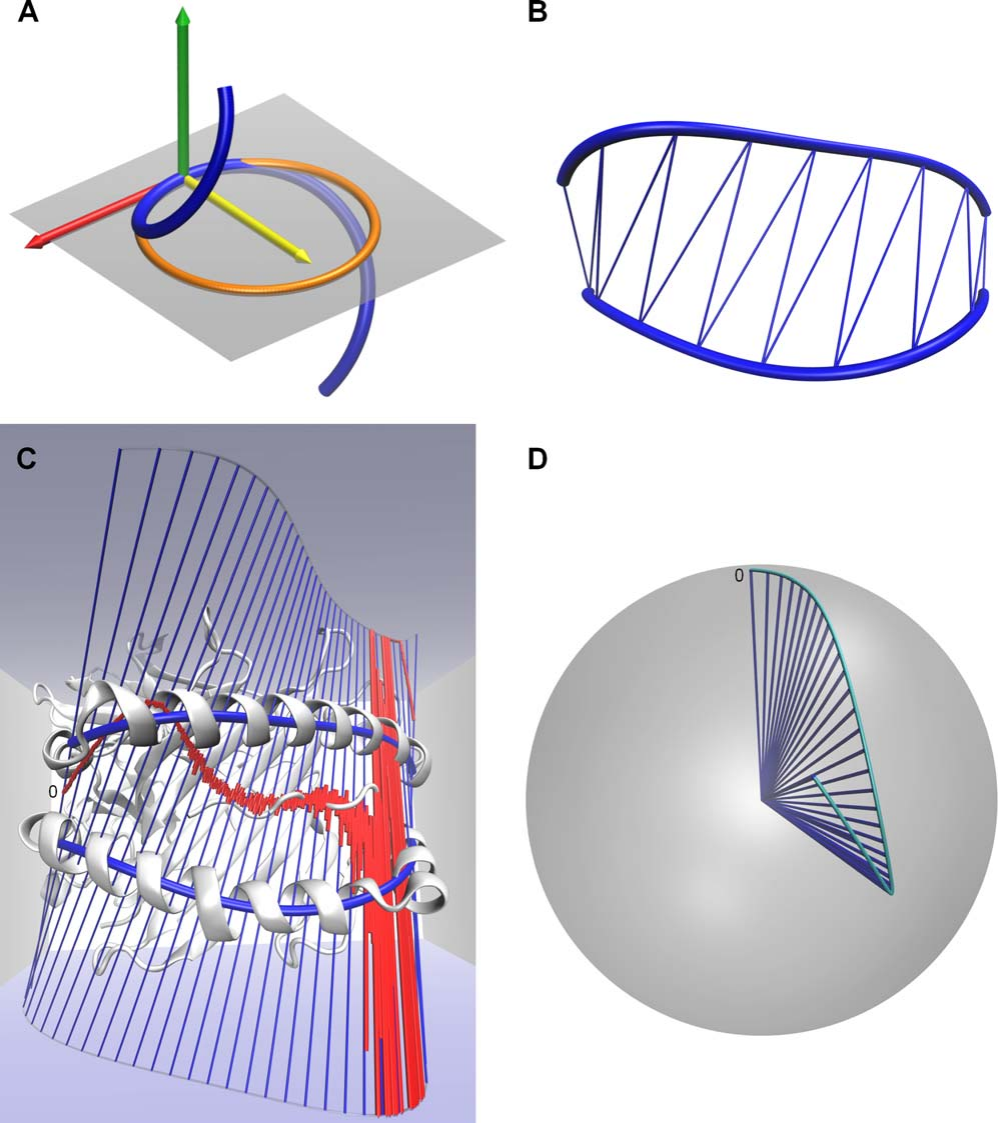
Differential geometric parameters for MH α-helices: (A) Representation of curvature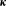 and torsion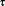: Curve (blue) with a local coordinate system in a curve point spanned by tangent vector (red), principal normal vector (yellow), and binormal vector (green). In the plane spanned by tangent vector and principal normal vector (gray), the circle of curvature (orange) is illustrated. For different views of this picture, we refer to Figure S1 of the Supporting Information. (B) Course-grained area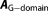: Two curves (blue) represent the two α-helices of the MH. We calculated the area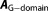 by a triangulation of the ruled surface between the two curves. For different views in a representative X-ray structure, we refer to Figure S2 of the Supporting Information. (C) Ruled surface generated by the curves (blue lines) representing the two α-helices of the MH H-2Kb (white) with the PDB accession code 1s7q (compare *Test set 2: MH1 cross evaluation*). The coarse-grained rulings (blue) originate from a movement of a straight line along the two curves. The striction curve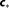 (red), representing the evolution of the distribution parameter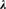, illustrates in a graphical way the skew parts (rulings are skew to each other) and the torsal parts (points of the striction curve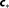 converges to infinity). For different views of this picture, we refer to Figure S3 of the Supporting Information. (D) Director cone (course-grained blue rulings fixed in origin) with the spherical curve (cyan) on the unit sphere. The conical curvature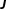 measures the curvature on the unit sphere of the spherical curve. The beginning of the ruled surface is marked with a zero. For different views of this picture, we refer to Figure S4 of the Supporting Information. Three-dimensional representations of this study were rendered in the software VMD.^23^ [Color figure can be viewed in the online issue, which is available at http://wileyonlinelibrary.com.]

The local curvature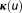 describes the local rate of change from the direction of the tangent vector. The inverse of the curvature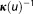 is the radius of the circle of curvature (Figure 1A). The local curvature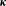 describes how strong the α-helices are curved in a certain range.

In three-dimensional space, the local *torsion*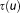,


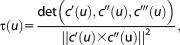
(2)

describes the local rate of change of the curve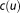 from the plane spanned by tangent and principal normal vector (Figure 1A). The local torsion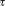 describes how strong the curves representing the α-helices are twisted in a certain range. In detail, the torsion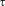 describes the rate of the twisting of the binormal vector about the axis spanned by the tangent vector. The sign of the torsion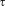 determines the direction of rotation of the binormal vector. A positive torsion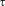 characterizes an anti-clockwise rotation, which we refer to as *right-handed twisting* of a curve. A negative torsion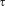 characterizes a clockwise rotation, which we refer to as *left-handed twisting* of a curve. Note that, these twisting properties have no bearing on the direction of the helical turns. We rather approximated the entire α-helices by curves and hence the direction of the helical turns is vanished in our model. The curvature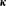 and the torsion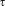 are able to uniquely describe a curve in three-dimensional space up to rigid body motions (e.g., parallel translations, rotations).

For a better biological interpretation, we took the moving average of the local curvature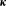 and local torsion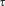 over four amino acids (AA) (corresponding to approximately one helical turn). With this method, we were able to investigate, which helical turns are responsible for a change in the parameters derived from the curve. For example, in the case of the curvature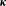, we obtained a value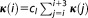 for each window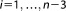, where the coefficient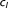 normalizes the value to the length of the curve and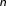 is the number of AAs of the considered α-helix. In the following, we will call these obtained values *average curvature* and *average torsion*, respectively.

### Area between helices

The curvature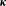 and torsion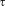 defined by eqs. (1) and (2) are measures for the spatial evolution of a single α-helix. In this subsection, we introduced a measure of the size of the G-domain. We approximated the area between two curves by introducing a triangulation of the ruled surface and computing the area of each triangle (Figure 1B). First, we added these areas, which lead to


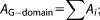
(3)

where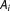 is the area of the *i*th triangle, and second, we normalized the area by the number of the AAs of both α-helices (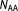) which leads to


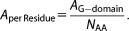
(4)

The area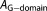 measures the absolute area of the G-domain. The area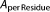 is a measure for the average area of the G-domain and is useful for the comparison of complexes with different number of AAs.

### Geometrical characterization of the ruled surface

Finally, we intended to describe the relative orientation of two α-helices; therefore, we introduced two characteristic parameters of the *ruled surface*.^24^ Generally, a ruled surface is used for the description of scattered data points originating from a surface in three-dimensional spaces.^25^ In our case, the ruled surface is employed as a kind of bridge between the two curves to describe the relative spatial alignment of the α-helices. A ruled surface results from a movement of a straight line along the two curves representing the two α-helices. For an approximation of this surface, we discretized both curves by the same number of curve points and connected the points of one curve with the corresponding points of the other curve (Figure 1C), yielding the so-called *generator lines* or *rulings* with direction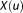. By calculating the foots of perpendicular of each pair of two adjacent rulings, we obtained a uniquely determined curve on the ruled surface, the so-called *striction curve*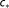 (red line in Figure 1C) with its *striction points*. Based on the striction curve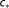, the parameterization of the ruled surface is given by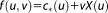, with the constraint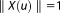. From this representation, we were able to calculate the two characteristic parameters of a ruled surface: The *distribution parameter*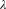 is defined as


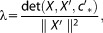
(5)

and the *conical curvature*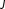 is given as


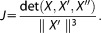
(6)

The distribution parameter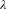 measures the change of the tangent plane along the rulings starting from the striction point. The absolute value of the distribution parameter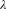 measures the velocity of the winding of the tangent plane around the rulings. A small value indicates a fast winding and a large value indicates a slow winding around the ruling. The sign of the distribution parameter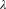 indicates the direction of the winding. The conical curvature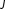 is defined as follows: The normed vectors of the rulings fixed in the origin span a so-called *director cone*. The *spherical curve*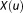 is obtained by the intersection of the director cone with the unit sphere (Figure 1D). The conical curvature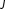 characterizes the change of the tangent of the spherical curve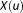. A ruled surface is called *conoidal*, if its director cone is a plane. In this case, the spherical curve is a great circle on the unit sphere and the conical curvature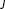 is zero. The sign of the conical curvature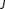 describes the direction of the curve on the sphere: A positive curvature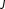 determines a left-hand bend on the sphere; a negative curvature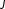 determines a right-hand bend on the sphere. The values of the conical curvature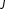 increase with decreasing radius for spherical curves which lay on small circles. Hence, the values of the conical curvature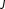 span a large range. For the visualization, we introduced two methods. First, the conical curvature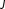 is depicted by zooming at different scales, which has the advantage that one is able to investigate the conical curvature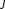 of different small circles. Second, we applied a nonlinear transformation to the conical curvature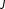 to map the values to an interpretable range. Taking the logarithm would cross one′s mind; however, the logarithm is only defined in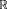 for positive values. Since the conical curvature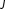 could be negative, the logarithm is not appropriate to scale our data. Instead, we applied the arc tangent function, which maps the conical curvature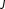 to the range of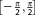. Additionally, these parameters (distribution parameter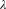, conical curvature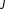) discriminate between *skew ruled surfaces* and *torsal ruled surfaces*. Skew ruled surfaces have skew rulings, whereas torsal ruled surfaces have either parallel rulings or the rulings have a common intersection. The ruled surface depicted in Figure 1C has both parts. For the major part of the ruled surface, the rulings are skew and hence we were able to calculate the striction points for the striction curve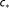. In the torsal part, the calculation of the striction points becomes numerically unstable, since the rulings lie practically parallel to each other. The torsal surface of Figure 1C is the range, where the surface changes its winding (see Results section). The distribution parameter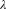 becomes zero in this part, since the vectors lie parallel to each other.

Also for the distribution parameter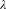 and the conical curvature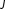, we computed the moving average over an average turn and, therefore, we obtained 36 average distribution parameters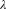 and average conical curvature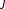, respectively, of the ruled surface for the MH1 case and 34 for the MH2 case. The AAs corresponding to the positions along the striction curve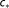 are listed in Supporting Information Table S1 for MH1 and Supporting Information Table S2 for MH2. In the following, we will call this obtained values *average distribution parameter* or *average conical curvature*, respectively.

### Treatment of the helical ends

At the end of secondary structure elements, some AAs may not be unambiguously classifiable. Therefore, an α-helix faces the problem of ambiguous boundaries.^26^ In order to avoid artifacts, we smoothed the curves and refrained from analysis of the last helical turn of each α-helix.

### Employed test sets

To test our developed methodology, we selected four test sets of X-ray structures from the Protein Data Bank (PDB).^27^

To make the results of the single X-ray structures comparable we superimposed each complex on the protein with the PDB accession code 1a1m. We chose this complex, since it is the first complex in alphabetical order of the PDB accession codes. Superimposition of all complexes to the same reference structure guarantees that the orientation of the local coordinate system of each complex points in the same direction.

Our parameters uniquely describe a curve and a ruled surface in three-dimensional space up to rigid body motions. Therefore, the only effect of choosing a different reference for superimposition would be a possible change in the sign of the torsion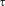, distribution parameter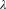, and/or conical curvature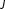.

Each of our test sets is described in the subsequent sections.

### Test set 1: How TRs deform MH α-helices

The aim of the first test set is to determine how the docking of a TR to pMH influences the α-helices and the shape of the G-domain. Based on the IMGT,^28^ we extracted all available (TR)/pMH from the PDB and classified the 403 complexes into pMH1 (321 complexes), pMH2 (18 complexes), TR/pMH1 (52 complexes), and TR/pMH2 (ten complexes). We compared the differences in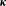,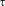,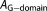,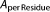,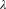, and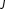 between the groups pMH1 and TR/pMH1 as well as between pMH2 and TR/pMH2. This test case is referred to as *Test set 1: How TRs deform MH* α-*helices*.

### Test set 2: MH1 cross evaluation

In our second example, we investigated two MH1 molecules (H-2Kb and H-2Db) in complex with four different peptides (KAVYNFATM, KAVYNLATM, KALYNFATM, KAVFNFATM) available by the PDB accession codes 1s7q, 1s7r, 1s7s, 1s7t, 1s7u, 1s7v, 1s7w, 1s7x.^29^ This research will shed light on possible differences in the spatial alterations caused by MH alleles with the same peptide as well as different peptides bound to the same MH allele. This test case is referred to as *Test set 2: MH1 cross evaluation*.

### Test set 3: Different TRs

In the third test set, we selected a MH2 molecule (I-Ab) presenting the peptide FEAQKAKANKAVD in complex with two different TRs (YAe62 and B3K506). The X-ray structures of these TR/pMH2 complexes have the PDB accession codes 3c60 and 3c5z.^30^ The two TRs differ by 34 AA mutations out of 198 AAs in the α-chain (17%) and 17 mutations out of 236 in the β-chain (7%). Altogether they differ by 12% of all TR AAs. These mutations are exclusively localized in the variable regions of the chains, especially in the CDRs. This test case is referred to as *Test set 3: Different TRs*.

### Test set 4: Helical disruption during a Molecular Dynamics simulation

In our last example, we investigated a MD simulation of a modified X-ray structure of PDB accession code 1k2d (I-Au/MBP1-11 complex) as published previously.^6^ In this previous study, we described a helical disruption in the helix G-ALPHA (AA 19-23). Here, we investigated how this helix deformation becomes noticeable with the methods presented herein. We compared the initial configuration of the MH simulation at the time 0 ns with an average structure of the time between the 15th and the 22nd ns as previously described by Knapp et al.^6^ This test case is referred to as *Test set 4: Helical disruption during a Molecular Dynamics simulation*.

### Overlap analysis for groups of complexes

As the differential geometry parameters are not only used for the comparison of two complexes, but also for groups of more complexes, an additional description of how this can be done in an appropriate way is given subsequently.

**Figure 2:**
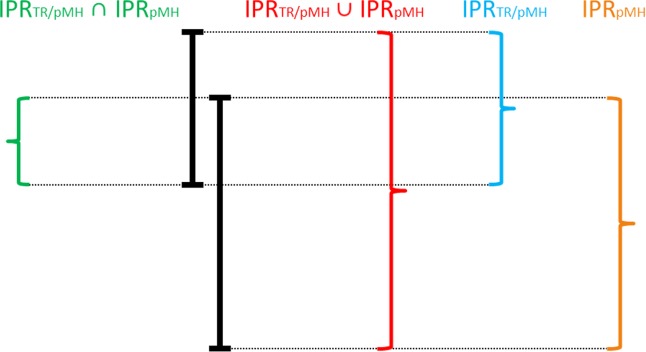
Percentages of overlap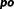: Percentages of overlap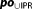 [eq. (7)] is obtained by the subset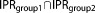 (green) relative to the union of the set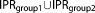 (red). Percentages of overlap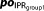 [eq. (8)] is obtained by the subset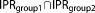 (green) relative to the subset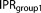 (blue). Percentages of overlap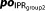 [eq. (9)] is obtained by the subset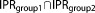 (green) relative to the subset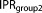 (orange). [Color figure can be viewed in the online issue, which is available at http://wileyonlinelibrary.com.]

For the comparison of two groups of complexes, we calculated for each position along each helix and ruled surface, respectively, the differential geometric parameters. We obtained in each position as many values for a parameter as number of complexes. For each position, we split the values according to its group membership, yielding two subgroups. Subsequently, we calculated the median and the boundaries of the 95%-interpercentile range (IPR) for each subgroup in each position. On this basis one is able to illustrate the developing of the median and the IPRs over the helix and ruled surface, respectively, by connecting the discrete values of each position with each other. This methodology is most appropriate; since several outliers are expected in the X-ray structures of the PDB^31^ and the group sizes differ. For a more detailed analysis, we calculated for each position the percentages of overlap,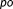, of the interception of the two groups (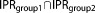). More precisely, it is possible to calculate the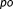 of the interception of the two groups (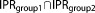) relative to the whole range, spanned by both groups (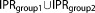), or relative to the one group (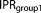), or relative to the other group (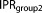). In the following, we described these three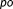 mathematically and illustrated them in Figure 2.

First, we computed the percentages of overlap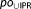 of the interception of the two groups (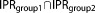) relative to the whole range, spanned by both groups (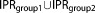)


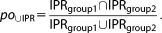
(7)

Second, we computed the percentages of overlap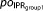 of the interception of the two groups (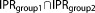) relative to the subset of one group (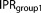)


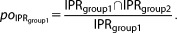
(8)

Third, we computed the percentages of overlap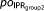 of the interception of the two groups (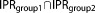) relative to the subset of the other group (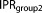)


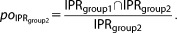
(9)

By these calculations, we obtained three percentage values in each position of the helix and the ruled surface, respectively, which describe the relative position of the IPRs to each other. Thus, we obtained for each position along a curve three percentage scores. Furthermore, we merged these percentage scores in a super-score, which is classified in three groups. The first group contained all positions of a curve or ruled surface, respectively, where all three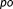 are <90%, the second group contained all positions, where two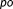 are <90%, and the third group contained all positions, where two or all three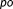 ≥90%. Therefore, each position within the curves or the ruled surface belongs to one group. We calculated the sum of the lengths of the IPRs (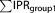,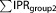) in the obtained groups as a scatter measure for the comparison between the two subgroups in interesting ranges of the curves and ruled surface, respectively.

## Results

In this section, we present the evaluation of our four test sets. For each complex (coordinates in nm), we calculated the six above described parameters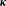 (nm^−1^) and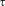 (nm^−1^) independently for each α-helix and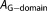 (nm^2^),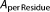 (nm^2^),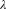 (nm), and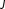 (–) once for each complex.

### Test set 1: How TRs deform MH α-helices

In this test set, we analyzed the differences in our parameters between pMH complexes bound to TRs and unliganded pMH complexes. Based on these calculations, we described the differences between pMH1 and TR/pMH1 as well as pMH2 and TR/pMH2 on the basis of the overlap analysis for groups of complexes (Methods).

**Figure 3:**
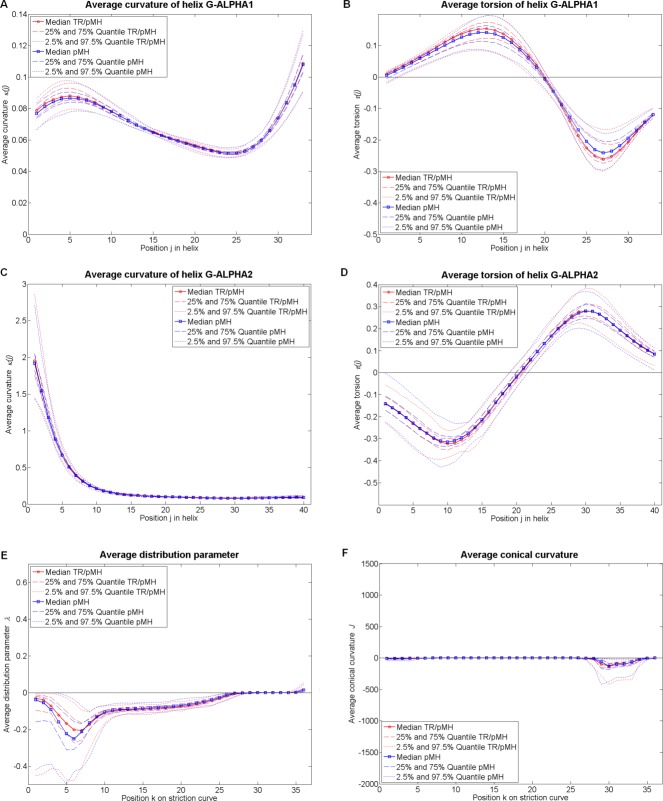
Results for Test set 1: How TRs deform MH α-helices, MH1 case. We compared 321 pMH1 complexes (blue) against 52 TR/pMH1 complexes (red). The medians are depicted as solid lines the interquartile ranges are depicted as dashed lines and the IPR are depicted as dotted lines. (A, B) Average curvature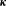 (nm^−1^) and average torsion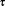 (nm^−1^) of the helix G-ALPHA1 at 34 (j) positions obtained as moving average of the local parameters over a turn (see Methods). (C, D) Average curvature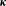 (nm^−1^) and average torsion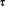 (nm^−1^) of the helix G-ALPHA2 at 40 (j) positions, obtained as moving average of the local parameters over a turn (see Methods). (E, F) Average distribution parameter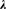 (nm) and average conical curvature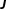 (–) of the ruled surface at 36 (k) positions on the striction curve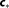, obtained as moving average of the local parameters over an average turn (see Methods). The zoomed figures of the average conical curvature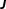 are depicted in Supporting Information Figure S5 and the arc tangent representation in Supporting Information Figure S6A. [Color figure can be viewed in the online issue, which is available at http://wileyonlinelibrary.com.]

By applying our first method to all MH1 complexes (321 pMH1 and 52 TR/pMH1), we calculated the average curvature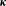 [eq. (1)] and the average torsion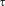 [eq. (2)] of the helix G-ALPHA1 and the helix G-ALPHA2. The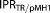 and the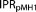, yielded by the overlap analysis, are depicted in Figures 3A and 3B for the helix G-ALPHA1 and in Figures 3C and 3D for the helix G-ALPHA2. In Table 1, we summarized our overlap analysis with the three percentages scores [eqs. 7 8 9] along the curves and ruled surface, respectively, colored according as the three groups of the super-scores. The helix G-ALPHA1 differs in the average curvature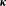 in the range from positions 19–23 (AA 19–26) and in the torsion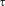 at the N-terminal end in the range from positions 1–10 (AA 1–13) and at the C-terminal end in the range from positions 27–32 (AA 27–35) (Table 1). The major differences of the helix G-ALPHA2 are found in the curvature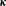 in the range from positions 5–7 (AA 5–10) and positions 24–29 (AA 24–32) as well as in the torsion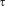 at the N-terminal end in the range from positions 1–14 (AA 1–17) (Table 1). In contrast to the helix G-ALPHA1, which has at the N-terminal end (positions 1–19) a right-handed twisting and at the C-terminal end (positions 20–33) a left-handed twisting, the twisting properties at the terminal ends of helix G-ALPHA2 are almost mirrored (Figures 3B and 3D), that is, parts of the curves, representing the α-helices, localized opposed having both either positive or both negative torsion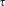 values.

**Table 1:** Overlap analysis between TR/pMH1 and pMH1: For each parameter, we calculated the three percentage scores *po*_∪IPR_ [eq. (7)],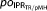 [eq. (8)], and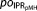 [eq. (9)] depicted in Figure 2

	Helix G-ALPHA 1						Helix G-ALPHA 2						Ruled surface					
			
	Average curvature (nm^−1^)			Average torsion (nm^−1^)			Average curvature (nm^−1^)			Average torsion (nm^−1^)			Average dist. par. (nm)			Average con. curvature (–)		
						
Pos.	*po*_∪IPR_, 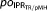 , 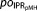	ΣIPR_TR/pMH_	ΣIPR_pMH_	*po*_∪IPR_, 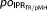 , 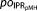	ΣIPR_TR/pMH_	ΣIPR_pMH_	*po*_∪IPR_, 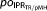 , 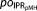	ΣIPR_TR/pMH_	ΣIPR_pMH_	*po*_∪IPR_, 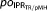 , 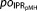	ΣIPR_TR/pMH_	ΣIPR_pMH_	*po*_∪IPR_, 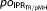 , 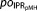	ΣIPR_TR/pMH_	ΣIPR_pMH_	*po*_∪IPR_, 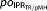 , 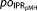	ΣIPR_TR/pMH_	ΣIPR_pMH_
1	85, 85, 100	0.0598	0.0537	83, 83, 100	0.5340	0.4450	88, 90, 98	1.4069	1.2814	75, 98, 76	2.2926	2.8038	93, 93, 99	2.1500	2.1343	60, 100, 60	103.9811	182.3447
2	86, 88, 98			82, 82, 100			78, 78, 100	1.8610	1.5301	76, 97, 78			89, 90, 99			59, 100, 59		
3	81, 88, 92			82, 82, 100			87, 87, 100			76, 95, 79			93, 93, 99			62, 100, 62		
4	**79, 89, 88**	**0.0190**	**0.0193**	81, 81, 100			78, 81, 95			78, 95, 82			91, 100, 91			50, 99, 50		
5	84, 92, 90	0.0846	0.0878	79, 79, 100			**71, 82, 84**	0.4921	0.4932	81, 96, 84			92, 100, 92			44, 98, 45		
6	91, 96, 94			80, 80, 100			**73, 86, 83**			83, 96, 86			85, 88, 95	0.4209	0.3899	63, 94, 65		
7	93, 100, 93			81, 81, 100			**72, 84, 83**			87, 100, 87			91, 100, 91	0.3083	0.3403	57, 95, 59		
8	98, 100, 98			84, 84, 100			81, 86, 94	0.0892	0.0817	79, 100, 80			81, 100, 81	0.6711	0.7932	86, 93, 91	1.3926	1.4022
9	96, 100, 96			86, 86, 100			91, 91, 100	0.2003	0.1906	63, 100, 63			82, 100, 82			84, 90, 92		
10	93, 100, 93			89, 89, 100			93, 93, 100			59, 100, 59			88, 100, 88			78, 92, 84	0.4004	0.4381
11	89, 100, 89	0.0206	0.0231	94, 94, 100	1.4719	1.4062	95, 97, 97			65, 100, 65			80, 100, 80			92, 98, 93	0.7684	0.8160
12	84, 99, 85			96, 96, 100			95, 98, 97			75, 100, 75			84, 99, 84			93, 100, 93		
13	82, 96, 85			97, 97, 100			89, 99, 89	0.1597	0.1909	82, 82, 99			85, 100, 85			81, 100, 81	2.0290	2.4793
14	98, 99, 99	0.0046	0.0046	97, 98, 99			86, 100, 86			77, 80, 96			80, 85, 93			81, 100, 81		
15	89, 100, 89	0.0179	0.0175	97, 98, 99			85, 100, 85			88, 92, 95	0.9357	0.9507	**77, 88, 86**	**0.1092**	**0.1131**	79, 100, 79		
16	82, 95, 86			94, 96, 98			80, 100, 80			90, 96, 93			**65, 81, 77**			81, 100, 81		
17	76, 82, 92			89, 92, 98			75, 100, 75			95, 100, 95			72, 93, 76	0.0451	0.0550	80, 100, 80		
18	73, 79, 90			89, 91, 98			81, 100, 81			99, 100, 99			**76, 87, 86**	**0.0505**	**0.0506**	88, 99, 89		
19	**70, 80, 86**	**0.0292**	**0.0295**	89, 91, 98			84, 100, 84			98, 98, 100			85, 98, 87	0.0480	0.0540	95, 100, 95	0.7683	0.7799
20	**68, 81, 81**			87, 90, 96			86, 100, 86			96, 97, 99			**63, 82, 73**	**0.0498**	**0.0566**	91, 95, 96		
21	**71, 84, 82**			96, 97, 99			98, 100, 98	0.0354	0.0350	98, 98, 99			71, 100, 71	0.0876	0.1137	89, 100, 89	4.1430	4.6340
22	**74, 88, 82**			92, 92, 100			96, 98, 98			94, 100, 94			77, 96, 80			86, 100, 86		
23	**78, 89, 86**			93, 93, 100			89, 92, 97			83, 100, 83	0.9217	1.0670	**76, 85, 88**	**0.0506**	**0.0490**	85, 100, 85		
24	80, 93, 85	0.0111	0.0123	96, 96, 100			**81, 89, 89**	0.0682	0.0687	85, 100, 85			97, 97, 100	0.1365	0.1321	70, 80, 85		
25	82, 97, 84			98, 98, 100			**74, 86, 84**			79, 100, 79			87, 93, 93			84, 100, 84		
26	90, 93, 97	0.0064	0.0061	92, 92, 100			**73, 85, 84**			89, 99, 90			88, 91, 97			94, 99, 95	4.9333	5.1232
27	83, 83, 100	0.0161	0.0138	88, 88, 100	0.5348	0.4679	**77, 86, 88**			87,100, 87			87, 87, 99	0.0332	0.0330	61, 61, 100	1988.2326	1872.1905
28	86, 87, 99			89, 89, 100			**79, 88, 88**			79, 100, 79			85, 100, 85			89, 89, 100		
29	91, 95, 96	0.1143	0.1213	86, 86, 100			**75, 89, 83**			83, 97, 85			58, 100, 58			68, 100, 68		
30	96, 99, 97			85, 85, 100			76, 93, 81	0.0267	0.0302	80, 91, 87			69, 100, 69			86, 86, 100		
31	90, 100, 90			87, 87, 100			83, 96, 86			82, 90, 90	0.3559	0.3519	79, 97, 81			80, 80, 100		
32	91, 100, 91			87, 89, 98			89, 97, 92	0.0171	0.0182	83, 90, 92			89, 99, 89			85, 85, 100		
33	92, 98, 94			90, 94, 96	0.0312	0.0304	88, 99, 89	0.1696	0.1927	82, 88, 92	0.1732	0.1651	94, 97, 97	0.0003	0.0003	75, 75, 100		
34							86, 100, 86			85, 91, 92	0.4278	0.4305	63, 64, 98	0.0768	0.0573	58, 98, 60		
35							86, 100, 86			85, 92, 91			56, 56, 99			96, 98, 97	21.7853	22.0348
36							82, 100, 82			84, 92, 90			80, 81, 99			81, 100, 81	2.8521	3.5230
37							86, 100, 86			84, 94, 89	0.2988	0.3086						
38							84, 96, 87			85, 97, 87								
39							82, 93, 88			84, 99, 84								
40							83, 91, 91	0.0391	0.0391	80, 100, 80								
Σ		0.3836	0.3890		2.5719	2.3495		4.5653	4.1518		5.4057	6. 0776		4.2379	4.3724		2131.2861	2095.7657

The results are highlighted according to the super-score (1st group: gray and bold, 2nd group: gray, and 3rd group: white). We calculated the sum of the IPRs in the obtained ranges and declared the overall sum in the last row. (We quoted four decimal places, since three decimal places are significant and the fourth decimal place contains the round-off error.)

With our second method, we calculated the area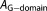 and the normalized area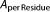 [see eqs. (3) and (4)] between the helix G-ALPHA1 and the helix G-ALPHA2 without considering the last three AAs to avoid artifacts. The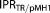 and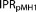 of the area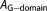 and the area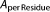 (see Table 2) overlap each other by 59%. The area of the G-domain of TR/pMH1 complexes is slightly bigger.

**Table 2:** Statistic of the area A_G–domain_ (nm^2^) and the area A_per Residue_ (nm^2^) for MH1 to analyze TR/pMH1 complexes against pMH1 complexes

	A_G–domain_ (nm^2^) of TR/pMH1	A_G–domain_ (nm^2^) of pMH1	A_per Residue_ (nm^2^) of TR/pMH1	A_per Residue_ (nm^2^) of pMH1
2.5% quartile	6.3447	6.2203	0.0869	0.0852
25% quartile	6.4502	6.4322	0.0883	0.0881
Median	6.5234	6.4926	0.0894	0.0889
75% quartile	6.5925	6.5770	0.0903	0.0901
97.5% quartile	6.8778	6.7322	0.0942	0.0922
IPR	0.5331	0.5119	0.0073	0.0070

We quoted four decimal places, since three decimal places are significant and the fourth decimal place contains the round-off error.

Characterizing the G-domain of the MH1 complexes, we applied our third method and calculated the striction curve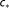 of the ruled surfaces with its two characteristic properties; the distribution parameter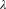 [see eq. (5)] and the conical curvature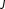 [see eq. (6)]. The results are illustrated in Figure 3E for the distribution parameter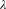 and in Figure 3F for the conical curvature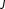. As we mentioned in Methods section, the conical curvatures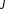 span a large range. Hence, we first zoomed into Figure 3F two times (Figures S5A, S5C, S5E of the Supporting Information) and second applied the arc tangent transformation (Figure S6A of the Supporting Information). Table 1 shows that the ruled surfaces of the two MH1 groups differ in the distribution parameter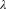 in the range from positions 8–23 (for the corresponding AAs see Supporting Information Table S1) and in the conical curvature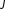 in the range from positions 1–7 as well as in the range from positions 27–34. The ruled surface has a negative winding in the range from positions 1–26, it becomes torsal (λ = 0 nm) in the range from positions 27–34 and has a positive winding in the range from positions 25–36 (Figure 3E). The spherical curve is a right-hand bend in the range from positions 1–10 (sign of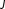 is negative), a left-hand bend in the range from positions 11–24 (sign of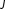 is positive), and finally again a right-hand bend in the range from positions 26–36 (sign of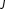 is negative) (Figure 3F and Supporting Information Figure S5E).

**Figure 4:**
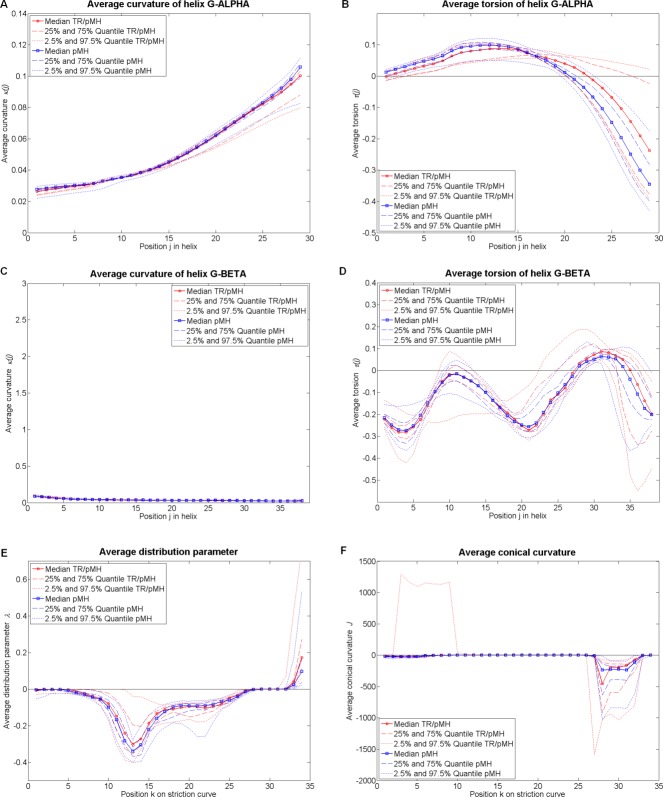
Results for Test set 1: How TRs deform MH α-helices, MH2 case. We compared 18 pMH2 complexes (blue) against 10 TR/pMH2 complexes (red). The medians are depicted as solid lines the interquartile ranges are depicted as dashed lines and the IPR are depicted as dotted lines. (A, B) Average curvature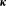 (nm^−1^) and average torsion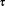 (nm^−1^) of the helix G-ALPHA at 29 (j) positions obtained as moving average of the local parameters over a turn (see Methods). (C, D) Average curvature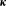 (nm^−1^) and average torsion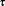 (nm^−1^) of the helix G-BETA at 38 (j), obtained as moving average of the local parameters over a turn (see Methods). (E, F) Average distribution parameter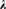 (nm) and average conical curvature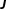 (–) of the ruled surface at 34 (k) positions on the striction curve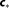, obtained as moving average of the local parameters over an average turn (see Methods). The zoomed figures of the average conical curvature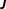 are depicted in Supporting Information Figure S5 and the arc tangent representation in Supporting Information Figure S6B. [Color figure can be viewed in the online issue, which is available at http://wileyonlinelibrary.com.]

Similar to the case of the MH1 complexes, we applied our parameters (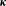 and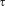 independently for each α-helix and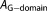,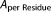,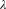, and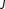 once for each complex) to all MH2 complexes of our Test set 1 (18 pMH2 and 10 TR/pMH2). We illustrated in Figures 4A and 4B, the curvature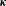 and the torsion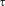 of the helix G-ALPHA and in Figures 4C and 4D, the curvature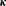 and torsion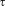 of the helix G-BETA. The two characteristics (distribution parameter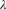 and conical curvature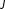) of the ruled surface are depicted in Figures 4E and 4F. We zoomed again two times in Figure 4F of the conical curvature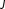 (Figures S5B, S5D and S5F of the Supporting Information) and transformed it with the arc tangent (Figure S6B of the Supporting Information). In Table 3, we illustrated the overlap analysis with the three percentages scores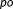 [eqs. 7 8 9] along the curves and ruled surface, respectively, colored according as the three groups of the super-scores of the MH2 complexes.

**Table 3:** Overlap analysis between TR/pMH2 and pMH2: For each parameter, we calculated the three percentage scores *po*_∪IPR_ [eq. (7)],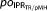 [eq. (8)], and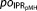 [eq. (9)] depicted in Figure 2

	Helix G-ALPHA						Helix G-BETA						Ruled surface					
			
	Average curvature (nm^−1^)			Average torsion (nm^−1^)			Average curvature (nm^−1^)			Average torsion (nm^−1^)			Average dist. par. (nm)			Average con. curvature (–)		
						
Pos.	*po*_∪IPR_, 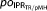 , 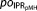	ΣIPR_TR/pMH_	ΣIPR_pMH_	*po*_∪IPR_, 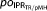 , 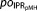	ΣIPR_TR/pMH_	ΣIPR_pMH_	*po*_∪IPR_, 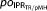 , 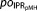	ΣIPR_TR/pMH_	ΣIPR_pMH_	*po*_∪IPR_, 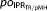 , 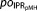	ΣIPR_TR/pMH_	ΣIPR_pMH_	*po*_∪IPR_, 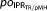 , 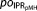	ΣIPR_TR/pMH_	ΣIPR_pMH_	*po*_∪IPR_, 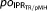 , 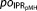	ΣIPR_TR/pMH_	ΣIPR_pMH_
1	52, 100, 52	0.0224	0.0466	85, 85, 100	0.7992	0.8767	86, 89, 97	0.0351	0.0321	79, 79, 100	0.5437	0.4363	53, 98, 53	0.0537	0.0938	**63, 75, 80**	**7192.7912**	**242.0003**
2	51, 100, 51			81, 81, 100			87, 92, 95	0.028	0.0269	78, 78, 100			59, 98, 60			**42, 58, 60**		
3	49, 100, 49			77, 78, 99			73, 73, 100	0.1603	0.0987	73, 77, 93			93, 93, 100	0.0698	0.0681	**2, 2, 77**		
4	47, 100, 47			74, 75, 98			55, 55, 100			**65, 75, 83**	**0.5008**	**0.5179**	98, 98, 100			**3, 3, 87**		
5	45, 100, 45			71, 73, 96			55, 55, 100			**57, 75, 71**			96, 99, 97			**3, 3, 98**		
6	44, 100, 44			70, 73, 95			57, 57, 100			**59, 85, 66**			83, 99, 83	0.0263	0.0312	**2, 2, 100**		
7	45, 100, 45			78, 82, 94			60, 60, 100			48, 50, 91	2.0719	1.2935	89, 99, 90	0.0370	0.0406	**1, 1, 92**		
8	**26, 72, 29**	**0.0099**	**0.0199**	86, 92, 93			65, 65, 100			35, 35, 100			**72, 88, 80**	**0.0501**	**0.0555**	**1, 1, 99**		
9	**21, 58, 25**			81, 91, 88			72, 73, 98			39, 39, 100			81, 82, 98	1.3196	0.7605	1, 1, 88	1231.3657	8.7009
10	**32, 67, 37**			74, 90, 81			**53, 72, 68**	**0.0254**	**0.0265**	45, 45, 100			70, 70, 100			5, 5, 90		
11	**32, 64, 39**			75, 94, 79			**54, 76, 64**			57, 57, 100			70, 70, 100			9, 9, 96		
12	**42, 80, 47**			76, 99, 77			**72, 80, 88**			64, 64, 100			59, 59, 100			**8, 8, 85,**	**7.3921**	**1.4899**
13	58, 100, 58	0.0089	0.0129	73, 100, 73			79, 79, 100	0.0623	0.0481	71, 71, 100			34, 34, 100			**28, 33, 64**		
14	66, 100, 66			69, 100, 69			79, 79, 100			71, 75, 94			**36, 44, 64**	**0.5815**	**0.5131**	**52, 73, 65**		
15	79, 100, 79			63, 100, 63			78, 78, 100			74, 81, 90			**36, 53, 54**			80, 80, 100	5.9707	3.8108
16	91, 100, 91	0.0112	0.0116	58, 100, 58			79, 79, 100			87, 100, 87			**57, 80, 66**			63, 63, 100		
17	86, 92, 93			54, 96, 55			79, 79, 100			60, 100, 60			78, 100, 78	0.7831	1.1470	53, 53, 100		
18	81, 87, 93	0.0163	0.0152	**65, 87, 72**	**2.3438**	**1.7700**	77, 77, 100			**58, 65, 85**	**0.2331**	**0.1365**	86, 100, 86			46, 46, 100		
19	75, 83, 90			**70, 82, 83**			73, 73, 100			**36, 39, 81**			81, 100, 81			46, 46, 100		
20	**71, 80, 87**	**0.1551**	**0.1555**	**66, 79, 80**			69, 69, 100			50, 51, 97	1.0263	0.6155	59, 100, 59			45, 45, 100		
21	**67, 77, 85**			**61, 73, 79**			**73, 81, 88**	**0.0053**	**0.0052**	58, 58, 100			55, 100, 55			44, 44, 100		
22	**64, 74, 83**			**57, 68, 77**			**58, 75, 72**			63, 64, 99			55, 100, 55			67, 67, 100		
23	**62, 72, 82**			**54, 65, 76**			64, 64, 100	0.0400	0.0293	56, 58, 93			69, 100, 69			73, 73, 100		
24	**58, 72, 75**			**52, 62, 76**			56, 56, 100			**48, 56, 78**	**0.5330**	**0.3836**	86, 100, 86			76, 100, 76		
25	**60, 75, 76**			**51, 60, 76**			63, 65, 96			**47, 55, 77**			77, 100, 77			63, 100, 63		
26	**64, 78, 79**			**51, 59, 78**			69, 70, 98			58, 58, 100	1.0473	0.6229	**64, 70, 88**	**0.0447**	**0.0356**	**41, 49, 72**	**1.9430**	**1.3216**
27	**68, 82, 81**			**52, 59, 82**			74, 77, 95			58, 58, 100			72, 96, 74	0.0246	0.0390	2, 2, 93	1513.3005	33.3980
28	**70, 85, 80**			**51, 56, 85**			87, 88, 99			62, 62, 100			21, 94, 21			93, 100, 93	937.5715	1011.3564
29	**70, 89, 76**			**48, 52, 85**			91, 100, 91	0.0066	0.0073	65, 65, 100			**50, 77, 59**	**0.0037**	**0.0012**	89, 89, 100	3.3865	2.8846
30							81, 100, 81	0.0060	0.0075	54, 54, 100			**17, 21, 49**			81, 81, 100		
31							91, 100, 91	0.0115	0.0121	**45, 52, 78**	**0.0901**	**0.0592**	**12, 12, 89**			89, 89, 100		
32							86, 92, 93			50, 90, 53	1.7403	1.7507	6, 6, 100	1.2107	0.6915	75, 75, 100		
33							86, 86, 100	0.0433	0.0620	60, 100, 60			37, 38, 93			9, 95, 9		
34							73, 73, 100			80, 100, 80			63, 66, 93			**36, 79, 40**	**0.9998**	**1.9758**
35							84, 93, 89			76, 80, 95								
36							54, 100, 54			62, 66, 91								
37							57, 100, 57			**52, 56, 88**	**0.9036**	**0.5611**						
38							65, 100, 65			**46, 50, 84**								
Σ		0.2238	0.2617		3.1430	2.647		0.4238	0.3557		8.6901	6.3772		4.2048	3.4771		10,894.7210	1306.9383

The results are highlighted according to the super-score (1st group: gray and bold, 2nd group: gray, and 3rd group: white). We calculated the sum of the IPRs in the obtained ranges and declared the overall sum in the last row. (We quoted four decimal places, since three decimal places are significant and the fourth decimal place contains the round-off error.)

The major differences of the helix G-ALPHA are found in the range from positions 18–29 (AA 18–32), especially in the torsion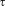, since pMH2 complexes have a more left-handed twisting in this range than TR/pMH2 complexes (Figure 4B). In the same way, the most differences of the helix G-BETA are found in the torsion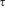 (Figure 4D). The helix G-ALPHA has at the N-terminal end a right-handed twisting and at the C-terminal end a left-handed twisting (Figure 4B), additionally, the results show differences in the twisting properties between the pMH2 and TR\pMH2 complexes. The helix G-BETA has almost everywhere a left-handed twisting, only in the range from positions 28–34 (AA 28–37) it has a right-handed twisting (Figure 4D).

We analyzed the area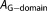 and the area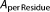 [see eqs. (3) and (4)] between the helix G-ALPHA and the helix G-BETA without considering the 3 AAs as in the case of the MH1 complexes. The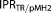 and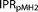 of the area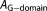 and the area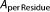 (see Table 4) overlap each other by 68%. The area of the G-domain of TR/pMH2 complexes is slightly smaller.

**Table 4:** Statistic of the area A_G–domain_ (nm^2^) and the area A_per Residue_ (nm^2^) for MH2 to analyze TR/pMH2 complexes against pMH2 complexes

	A_G–domain_ (nm^2^) of TR/pMH2	A_G–domain_ (nm^2^) of pMH2	A_per Residue_ (nm^2^) for TR/pMH2	A_per Residue_ (nm^2^) for pMH2
2.5% quartile	5.9055	6.0557	0.0881	0.0904
25% quartile	6.0713	6.3546	0.0906	0.0948
Median	6.3296	6.4153	0.0945	0.0958
75% quartile	6.4397	6.4510	0.0961	0.0963
97.5% quartile	6.6042	6.7035	0.0986	0.1001
IPR	0.6987	0.6478	0.0105	0.0097

We quoted four decimal places, since three decimal places are significant and the fourth decimal place contains the round-off error.

The major differences of the characteristic properties of the ruled surfaces between the MH2 groups are found in the distribution parameter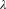 in the range from positions 8–16 (for the corresponding AAs see Supporting Information Table S2) and in the conical curvature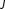 in the range from positions 1–14 (Table 3). In both cases, the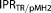 takes a larger range than the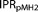. The ruled surface has a negative winding in the range from positions 1–27, it becomes torsal (λ = 0 nm) in the range from positions 28–32 and has a positive winding in the range from positions 34–35 (Figure 4E). The spherical curve is a right-hand bend in the range from positions 1–10 (sign of is negative), a left-hand bend in the range from positions 12–24 (sign of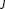 is positive), and finally again a right-hand bend in the range from positions 25–34 (sign of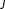 is negative) (Figure 4F and Supporting Information Figure S5F).

Figure 4F shows that there are one or more outlier complexes, that have a positive and large conical curvature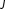 in the range from positions 1–10, which we found according the Hampel test.^32^ We picked out those complexes, which have an extreme value in more than two positions in at least one parameter (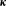,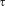,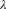,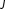,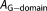, and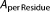) and displayed them in Table 5. On this basis, we found four TR/pMH1 complexes, 13 pMH1 complexes, and one TR/pMH2. For example, if one compares Figure 4E with Table 5, the outlier 3c60 is clearly visible as a peak in the range from positions 3–9.

**Table 5:** Outliers of Figure 3 and Figure 4

	Pdb-id	Parameter	Positions
TR/pMH1	1kj2	λ	1–5
	2esv	τ of the helix G-ALPHA2	29–31
	2f54	J	30–33
	3h9s	κ of the helix G-ALPHA2	1–3
pMH1	1jge	J	29–32
	1kj3	λ	1–6
	1l6q	κ of the helix G-ALPHA1	30–33
		τ of the helix G-ALPHA1	22–25
	1rjy	λ	1–3
	1rog	κ of the helix G-ALPHA1	32–33
		κ of the helix G-ALPHA2	1–2
		τ of the helix G-ALPHA2	28–36
	1roi	κ of the helix G-ALPHA2	1–3
		τ of the helix G-ALPHA2	1–8, 14–23, 29–40
	1rok	τ of the helix G-ALPHA2	28–39
	1rol	τ of the helix G-ALPHA1	4–17, 24–33
		τ of the helix G-ALPHA2	1–5, 10–21, 27–40
	1zt7	τ of the helix G-ALPHA2	1–5
		λ	1–4
	2bsr	J	29–32
	2c7v	J	29–32
	3h9h	τ of the helix G-ALPHA2	1–5
	3ixa	τ of the helix G-ALPHA2	1–9, 34–36
TR/pMH2	3c60	J	3–9, 27

Furthermore, we investigated to which extent the found differences originate from uncertainties in the X-ray structures. Therefore, we repeated the analysis and weighted our curves by the inverse of the b-factors. The relative differences between the parameters remain similar (Supporting Information Figure S7 for MH1 and Supporting Information Figure S8 for MH2). Additionally, we visualized the differences between the two types of curves graphically (Supporting Information Figure S9). In our software,1 we provide an additional option to weight the curves with the inverse of the b-factors.

One might ask if the differences in our parameters are also reflected in standard measurements. For this purpose, we calculated the RMSD-values between each complex and the average structure of all complexes (Figure S10 of the Supporting Information). The comparison of our method and the RMSD shows that both methods point to the same direction. Additionally, our method is able to analyze the differences in the underlying geometry and the relative spatially alignment of the α-helices.

### Test set 2: MH1 cross evaluation

**Figure 5:**
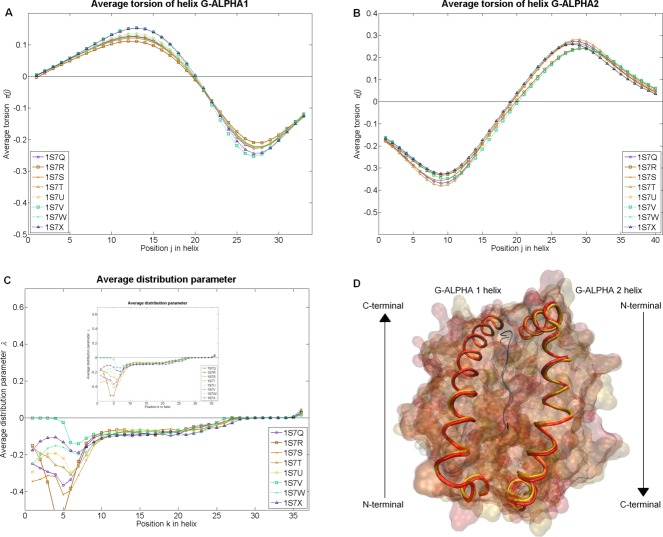
Results of Test set 2: MH1 cross evaluation. We compared eight pMH1 complexes (H-2Kb and H-2Db presenting four different peptides each). (A) Average torsion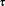 (nm^−1^) of the helix G-ALPHA1 at 34 (j) positions obtained as moving average of the local parameters over a turn. (B) Average torsion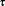 (nm^−1^) of the helix G-ALPHA2 at 40 (j) positions obtained as moving average of the local parameters over a turn. (C) Average distribution parameter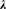 (nm) of the ruled surface at 36 (k) positions on the striction curve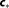 obtained as moving average of the local parameters over an average turn. (D) Graphical visualization of the H-2Kb (orange with the PDB accession code 1s7r) and the H-2Db (red with the PDB accession code 1s7v) in complex with the peptide KAVYNLATM. All parameters of this example are illustrated in Figure S11 of the Supporting Information. In Supporting Information Table S3, we presented the resolutions and b-factors of these complexes.

In the example *Test set 2: MH1 cross evaluation*, we analyzed the differences between MH1 types (H-2Kb and H-2Db) and four different bound peptides (KAVYNFATM, KAVYNLATM, KALYNFATM, and KAVFNFATM). The results show that the eight complexes are clustered according to their MH1 alleles (Figure 5, respectively, Supporting Information Figure S11): In this example, the four complexes of H-2Db (PDB accession codes 1s7u, 1s7v, 1s7w, 1s7x) show a slightly increased average curvature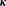 at the terminal ends of the helix G-ALPHA1 (Supporting Information Figure S11A). This cluster is also observable in the average torsion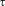 (Figure 5A), where the four H-2Db complexes have higher absolute average torsion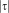 values, than the H-2Kb complexes (PDB accession codes 1s7q, 1s7r, 1s7s, 1s7t).

Another interesting case is the analysis of the peptide KAVYNLATM in complex with H-2Kb (1s7r) and in complex with H-2Db (1s7v), since the parameters (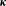,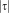) of the helix G-ALPHA1 are the lowest ones in the H-2Kb cluster and the highest ones in the H-2Db cluster. We observed similar behavior in the helix G-ALPHA2, where the two MH alleles in complex with the peptide KAVYNLATM occur as outliers (Figure 5B). The average distribution parameter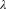 (Figure 5C) shows severe differences in the first part (positions 1–7), where the ruled surface of all complexes has a negative winding with exception of the H-2Db in complex with the peptide KAVYNLATM (1s7v), which is torsal (λ = 0 nm). The H-2Db complexes have a more negative winding than the H-2Kb complexes.

In Supporting Information Figure S11F and in the arc tangent representation in Supporting Information Figure S6C, respectively, the conical curvature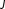 of the H-2Db in complex with the peptide KAVYNLATM (1s7v) becomes apparent as outlier in the range from positions 1–6, where the right-hand bend of the spherical curve is increasingly sharper. The smallest area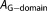 of the G-domain has the H-2Db in complex with the peptide KAVYNLATM (1s7v) with 6.3363 nm^2^. The H-2Db in complex with the peptide KAVFNFATM (1s7x) has the largest area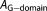 of the G-domain with 6.5270 nm^2^ (Table 6). In Figure 5D, we illustrated the two MH alleles in complex with the KAVYNLATM. As one can see, the helical backbones are not identical, but appear quite similar. Here, our methods are able to detect even subtle differences in the α-helices and the area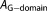 of the G-domain, which cannot be assessed with the naked eye.

**Table 6:** Area A_G–domain_ (nm^2^) and area A_per Residue_ (nm^2^) for the complexes of Test set 2: MH1 cross evaluation

	1s7q	1s7r	1s7s	1s7t	1s7u	1s7v	1s7w	1s7x
A_G–domain_	6.4091	6.4173	6.4166	6.4724	6.4697	6.3363	6.4924	6.5270
A_per Residue_	0.0878	0.0879	0.0879	0.0887	0.0886	0.0868	0.0889	0.0894

We quoted four decimal places, since three decimal places are significant and the fourth decimal place contains the round-off error.

The RMSD values between each complex and the average structure is illustrated in Table S4 of the Supporting Information.

### Test set 3: Different TRs

**Figure 6:**
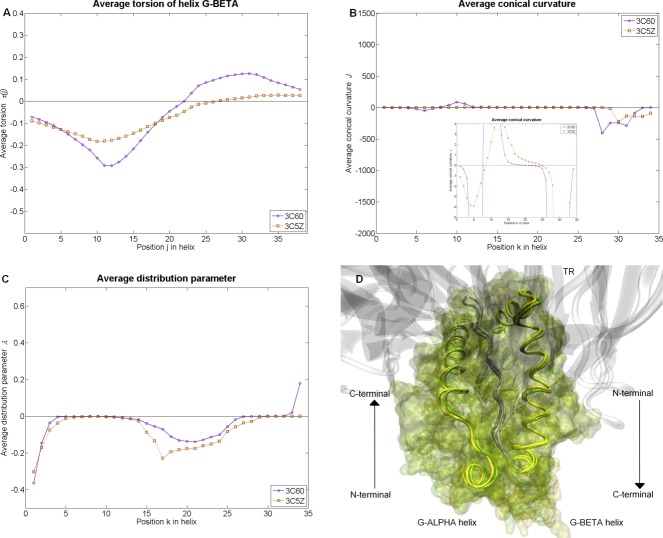
Results of Test set 3: Different TRs. We compared two TR/pMH2 complexes (I-Ab presenting the peptide FEAQKAKANKAVD in complex with the TR YAe62 and in complex with the TR B3K506). (A) Average torsion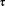 (nm^−1^) of the helix G-BETA at 38 (j) positions originating by calculating the moving average of the local parameters over a turn. (B, C) Average distribution parameter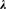 (nm) and average conical curvature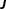 (–) of the ruled surface at 34 (k) positions on the striction curve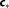, originated by calculating the moving average of the local parameters over an average turn. (D) Graphical visualization of the I-Ab in complex with YAe62 (yellow with the PDB accession code 3c60) and the I-Ab in complex with B3K506 (green with the PDB accession code 3c5z). All parameters of this test set are illustrated in Figure S12 of the Supporting Information. In Supporting Information Table S3, we presented the resolutions and b-factors of these complexes. [Color figure can be viewed in the online issue, which is available at http://wileyonlinelibrary.com.]

Interesting insights are found *Test set 3: different TRs*, where we investigated the I-Ab allele presenting the peptide FEAQKAKANKAVD in complex with the TR YAe62 (PDB accession code 3c60) and in complex with the TR B3K506 (PDB accession code 3c5z). The helix G-ALPHA shows very few differences between the two TR/pMH2 complexes (Supporting Information Figures S12A and S12B). In contrast, the helix G-BETA of the MH2 in complex with B3K506 (3c5z) has on average a 0.0137 nm^−1^ higher left-handed twisting at the N-terminal end (positions 1–4, corresponding to AA 1–7) (Figure 6A). In the range from positions 5–18 (AA 5–21), the MH2 in complex with the YAe62 (3c60) has an average value of 0.0551 nm^−1^ (Figure 6A). In the C-terminal end of the helix G-BETA (positions 19–38, corresponding to AA 19–41), the MH2 in complex with YAe62 (3c60) has, on average a 0.0711 nm^−1^ higher right-handed twisting (Figure 6A).

These alterations of the helix G-BETA influence the ruled surface and are reflected in the distribution parameter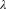 (Figure 6C). In the range of positions 2–4, the ruled surface of the MH2 in complex with the B3K506 (3c5z) has on average a 0.0330 nm a more negative winding, which increases to 0.0466 nm on average in the range from positions 14–28.

In the range of positions 33–44, the ruled surface of the MH2 in complex with the YAe62 (3c60) has a positive winding, whereas the MH2 in complex with the B3K506 (3c5z) is torsal (λ = 0 nm).

The spherical curve of the MH2 in complex with the YAe62 has in the range from positions 1–12 and 27–32 a higher conical curvature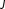 (Figure 6B). In the range from positions 13–26, the MH2 in complex with the B3K506 (3c5z) has about 0.9575 higher conical curvature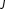. Both spherical curves bend to the right in the range from positions 1–7 and bend to the left afterwards. The one of the MH2 in complex with YAe62 (3c60) becomes a right-hand bend again at position 21, whereas the other at position 24. The area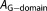 of the MH2 in complex with the YAe62 amounts to 5.8710 nm^2^ (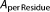 =0.0860 nm^2^). The area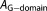 of the MH2 in complex with the B3K506 amounts to 5.7622 nm^2^ (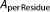 =0.0876 nm^2^).

The RMSD between the G-ALPHA helices is 0.0647 nm while it is 0.3976 nm between the G-BETA helices.

### Test set 4: Helical disruption during a Molecular Dynamics simulation

**Figure 7:**
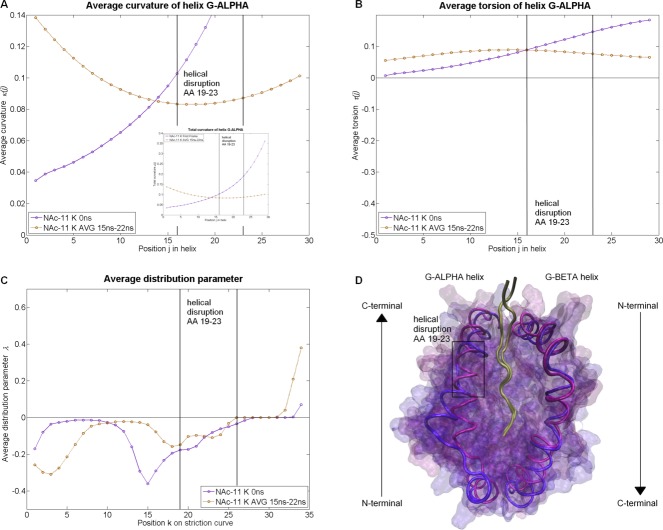
Results of Test set 4: Helical disruption during a Molecular Dynamics simulation. We compared two snapshots of the I-Au/MBP1-11 complex simulation (at time 0 ns and an average of the time between the 15th and the 22nd ns). (A, B) Average curvature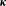 (nm^−1^) and average torsion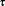 (nm^−1^) of the helix G-ALPHA at 29 (j) positions originated by calculating the moving average of the local parameters over a turn. (C) Average distribution parameter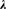 (nm) of the ruled surface at 34 (k) positions on the striction curve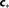, originated by calculating the moving average of the local parameters over an average turn. (D) Graphical visualization of the snapshot at 0 ns (violet) and the average snapshot (blue). All parameters of this test set are illustrated in Figure S13 of the Supporting Information. In Supporting Information Table S3, we presented the resolutions and b-factors of these complexes.

Employing our methods, we were able to investigate the impact of a helical disruption in a small region of the helix as well as on the whole G-domain. In our example, we compared the configuration at time 0 ns of the I-Au/MBP1-11 complex simulation, with the average configuration. In this average structure, the helix G-ALPHA loses its form between AA 19–23. The consequence of this helical disruption for the helix G-ALPHA in this range is a decrease by 0.0575 nm^−1^ of the curvature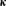 (Figure 7A) and a decrease by 0.0369 nm^−1^ of the torsion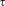 (Figure 7B). Hence, the geometry of the whole helix G-ALPHA is influenced by this helical disruption: The curvature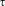 increases on average by 0.0515 nm^−1^ in the range from positions 1–13 (AA 1–16) and decreases by 0.0991 nm^−1^ in the range from positions 14–29 (AA 14–32). The torsion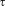 increases on average by 0.0369 nm^−1^ in the range from positions 1–15 (AA 1–18) and decreases to 0.0556 nm^−1^ in the range from positions 16–29 (AA 16–32). The helix G-ALPHA retains a right-handed twisting. The differences in the helix G-BETA are minor (Supporting Information Figures S13C and S13D).The biggest difference is a decrease in the torsion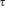 by 0.0180 nm^−1^ in the range from positions 16–38 (AA 16–41). The whole helix G-BETA has a left-handed twisting (Supporting Information Figure S13D).

These alterations of the helices influence the G-domain, characterized by the ruled surface, enormously (Figure 7C). The ruled surface has in the range of the helical disruption of the helix G-ALPHA a negative winding (positions 19–26). In the first part of this range (positions 19–22), the distribution parameter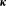 increases by 0.0409 nm, in the second part (positions 23–24) the distribution parameter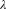 decreases by 0.0308 nm, and finally it increases by 0.0263 nm in the third part (positions 25–26). Hence, the distribution parameter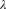 of the whole ruled surface is influences: It decreases by 0.1285 nm in the range from positions 1–10 and increases by 0.1188 nm in the range from positions 11–22. The helical disruption of the helix G-ALPHA influences the spherical curve (Supporting Information Figure S13F): The conical curvature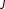 decreases in the first part (positions 19–24) by 1.202 and in the second part (positions 25–26) by 27.8118. Hence, the whole spherical curve is influenced by the helical disruption: The conical curvature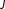 increases by 1.3786 in the range from positions 1–6, it decreases by 0.5364 in the range from positions 7–13, and it increases by 0.1889 in the range from positions 14–19. The spherical curve of both states of the complex is a right-hand bend from position 1. The changeovers from the right-hand bend to the left-hand bend changes from position 8 to position 10 during the simulation. It becomes again a right-hand bend at position 24, which changes to position 21 after the simulation. In Figure 7D, the MH2 complex at the time 0 ns and the average structure are illustrated graphically, where the obvious alterations are observable. The area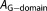 amounts 6.1573 nm^2^ (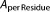
= 0.0919 nm^2^) and increases over time to 6.8840 nm^2^ (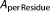 = 0.1027 nm^2^), this corresponds to an increase of 11%.

The RMSD between the two G-ALPHA helices is 0.4711 nm while it is 0.3641 nm between the G-BETA helices.

## Discussion

### Why are α-helices biologically important?

In this study, we demonstrated the application of several differential geometric parameters (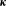,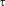,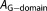,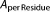,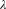, and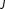) to characterize the alterations in MH α-helices. The importance of MH α-helices in T-cell activation and the importance of this process in human disease are manifold. It ranges from forming the classical G-domain for peptide presentation^1^ to several other functions as briefly summarized in the following.

There is evidence that indirect recognition of MH plays an important role in allograft rejection.^33^–^35^ Previous studies showed that peptides derived from α-helices of allo-MH antigens are potent inducers of CD4+ and CD8+ T cell responses after cardiac allograft rejection.^35^

It is also known that donor–recipient class II matching improves kidney graft survival in humans.^36^ Furthermore, T-lymphocytes from spleen showed strong proliferation to peptides from α-helical region of MH ten days after rejecting kidney allografts.^37^ Data from the same study also demonstrated that the physiological processing of donor antigens influence which MH peptides will be important in indirect allorecognition in transplantation. Moreover, the role of α-helices has been implicated in hematopoietic stem cell transplantation (SCT), which is an established treatment for a variety of hematological disorders, such as hematological malignancies. Complications of SCT from HLA-mismatched donors are often graft-versus-host disease or graft failure.

While it is generally believed that the motifs involved in TR/MH association are predominantly located in the α-helices of the MH^38^ the total number of AA substitutions in the MH not necessarily correlates with alloreactivity^39^ However, predictive methods for HLA matching often attach greater significance to AAs of the MH α-helices^40^ suggesting a recognition code in the side-chain orientation of the MH α-helices.^41^

Activation of T cells by MH molecules has also found its application in the field of oncology. Development of MH2 vaccines is a promising approach for the prevention and treatment of patients with metastatic cancers.^42^ The MH2 vaccines are tumor cells that are genetically modified to express MH2 and co-stimulatory molecules and are capable to activate tumor-reactive CD4+ T lymphocytes.

Further, superantigens do not bind inside the G-domain but rather interact with the outside, that is, the MH α-helices.^43^,^44^ Especially, in the case of superantigen staphylococcal enterotoxin A interaction both α-helices along the G-domain are required for superantigen induced T cell proliferation.^45^

Other hypotheses on the importance of the MH α-helices even take one step further and postulate that the evolutionary-conserved, tri-dimensional cusp found in the middle of the MH α-helices, may play an important role in a variety of biologic functions which have little to do with classical antigen presentation:^46^ Examples for such extended functions of MH could be hemochromatosis,^47^ association with pheromone receptors,^48^ or association with IgG transport.^49^

### Biophysical mechanism of the ligand induced conformational changes on MH α-helices

It is known that MH1 helices move slightly when adjusting to peptide ligands and TRs.^1^ Even subtle changes in the environment of the peptide can affect thermostability and alloreactivity.^50^ Recently, the conformational variations in MH2 structures have been broadly reviewed:^51^ While the canonical conformation of the peptide binding mode is maintained by a network of hydrogen-bonds between the peptide backbone and the MH, the polymorphic sequence of MH2 molecules causes differences in the architecture and charge of the MH2 structure. This affects especially the regions of the MH G-domain with its binding pockets for anchoring the presented peptide resulting in severe differences in the peptide binding capability. According to Painter and Stern,^51^ these structural alterations in the helices are especially present in the helical regions α45–54 (AA 1–9 of helix G-ALPHA) and in the kink in the helical region β62–71 (AA 11–20 of helix G-BETA). The later site was also reported as undergoing peptide induced conformational changes: In some outliers, the kinked region even tilted toward the peptide G-domain by about 4 Å. Further experimental evidence for peptide-induced conformational changes was found in Lys α67, Arg α50, Lys β98, and Arg β189.^52^ Interestingly, no X-ray or NMR structure has been reported for MH2 in absence of a peptide. This indicates that the peptide has severe effects on the stability of the MH in general and especially on the α-helices around the peptide. Hence, computational groups have performed MD simulations to investigate the behavior of MH2 in the absence of a peptide and found severe structural changes in the whole peptide G-domain^53^ up to unfolding of a helix part and adopting a peptide-like binding mode to MH.^52^ Apart from classical MH molecules, there is a plethora of MH-like structures consisting of two semiparallel α-helices and a β-sheet floor below.^54^ This illustrates the high flexibility and adoptability of MH-like folds.

### Differential geometric parameters in previous protein structure studies

Differential geometry parameters are well-known in the field of computational molecular biology, where they are mainly used for calculations involving solid-state NMR and protein folding. Mainly, the torsion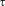 is applied in the field of structure determination of proteins using orientational restraints and dynamics to determine protein structures from solid-state NMR.^55^,^56^ Bates et al.^57^ presented a method about the theoretical modeling of biomolecules. They calculated the minimal molecular surface by a mean curvature minimization algorithm. Koh and Kim 58 used the mean curvature to show that the β-sheets in proteins are minimal surfaces. In another study, Koh et al.^59^ used the mean curvature to investigate the stability of a residue in a β-sheet. Ranganathan et al.^26^ introduced the automated protein structure analysis method based on the calculation of curvature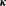 and torsion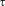 to describe and categorize the conformational features of proteins based on the backbone of the protein. In comparison to previous studies which calculated the curvature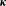 and the torsion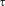 of the secondary structure (e.g., Ref. 26), we represented the α-helices by curves which are used for the calculations. The advantage is that we interpreted the α-helices as a whole object element and investigated the alterations of the walls of the G-domain and not of the periodical helical turns. Employing differential geometry for investigating MH α-helices is a novel approach designed specifically for the characterization of alterations in (TR)/pMH interactions. On the one hand, the method offers the opportunity to analyze subtle changes; on the other hand, obvious changes can be described quantitatively. Our method enables to handle spatial alterations in a structured way, without ambiguous interpretation of a visualization of the molecule. Furthermore, parts of this approach are in principle applicable to helical structures of other protein structures as shown in Supporting Information Figure S14. Our method is also applicable for other types of secondary structures, for example, π-helices, 3_10_-helices, and β-sheets. The only restriction for using this method is the number of atoms, which are used for constructing the curve, since the degree of the polynomial can only be as high as the number of atoms minus 1.

### Differences between pMH and TR/pMH complexes

An aspect of this study was to characterize the differences between pMH bound to a TR and pMH complexes not bound to a TR. As TRs have about the same size as MHs one would expect that the docking of a TR would induce severe conformational changes in the MH. However, only subtle differences in the mean of the parameters (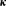,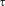,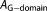,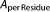,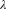, and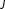) between liganded and unliganded pMH complexes could be observed for MH1 (Figure 3) and MH2 (Figure 4). This finding is in agreement with the visual impression of the comparison between the X-ray of HLA-B*08:01^10^ and HLA-B*08:01 bound to the LC13 TR,^60^ which also shows only little effect as the MH α-helices.

Additionally, the provided IPR of Figures 3 and 4 allow scientists to determine if a new resolved MH complex has extreme properties regarding the geometric parameters (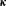,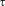,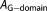,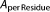,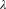, and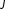). This is especially useful, because many published X-ray structures represent extreme configurations and are therefore not a representative state of the natural configuration.^31^

### Mirroring of torsion between MH1 α-helices

An interesting observation is that the torsion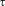 of the two MH1 α-helices is almost mirrored (compare Figures 3B and 3D): Parts of the helix G-ALPHA1 and helix G-ALPHA2 which are adjacent to the same peptide residue show similar twisting properties. In the MH2 class, such a phenomenon is not observable. An explanation could be that the spatial rearrangements of MH1 α-helices in contrast to MH2 helices are restricted by the closed G-domain and therefore a bulky residue pushes apart the MH helices on both sides.

### Effect of TR binding on areas between α-helices in MH1 and MH2

Our analysis of the data (Tables 2 and 4) shows a trend for the area spanned by the MH α-helices: The binding of a TR to MH1 increases the area while the binding of a TR to MH2 decreases the area.

### Most flexible parts in α-helices

Our results suggest that parts of the α-helices show a tendency to have more spatial alterations than other parts. We observed the most flexible parts of the helix G-ALPHA1 at the terminal ends (AA 1–5 and AA 28–35, Figure 3A), as well as AA 11–19 and AA 23–32 (Figure 3B). The helix G-ALPHA2 exhibits the biggest differences at the N-terminal end (AA 1–15, Figures 3C and 3D), as well as at the C-terminal end (AA 27–43, Figure 3D). The largest variance of the parameters of the ruled surface, describing the arrangement of the α-helices to each other, are found between AA 1–12 and AA 26–32 of helix G-ALPHA1 and AA 6–14 and AA 26–41 of helix G-ALPHA2 (Figures 3E and 3F). In the MH2 class, we found the biggest variance of the helix G-ALPHA at AA 20–32 (Figures 4A and 4B). The helix G-BETA is very flexible, especially at AA 7–19 and the C-terminal end at AA 32–41 (Figures 4C and 4D). The largest variance of the parameters of the ruled surface are found between AA 1–10 and AA 24–31 of the helix G-ALPHA and AA 2–11 and AA 19–41 of the helix G-BETA (Figures 4E and 4F).

### Outlier complexes identified by our analysis

For almost all complexes of an MH class, the parameters (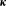,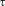,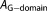,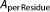,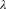, and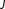) were quite similar. Nevertheless, Table 5 illustrates some outliers, which behave differently. For example, the TR/pMH2 complex (PDB accession code 3c60) has a severely increased conical curvature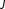.

### Clustering of MH1 alleles independent of presented peptide

Clustering of the calculated parameters of the example *Test set 2: MH1 cross evaluation* shows that each of the two MH1 types (H-2Kb and H-2Db) loaded with four different peptides forms a cluster with regard to the curvature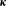 and torsion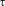 of the helix G-ALPHA1. This is remarkable because the α-helices in the graphical visualization make a very similar impression (Figure 5D) and the alterations in the α-helices are known to be subtle.^4^ For example, Miller et al.^61^ showed that retention of the peptide and a single mutation in the MH complex can cause an altered conformation of the CDR3α loop. On this basis, the shape of the G-domain is likely to influence the CDR. Simultaneously, our test set shows that based on our method, the impact of different peptides on the α-helices and the G-domain can be analyzed as well (e.g., the peptide outlier KAVYNLATM).

### Binding of a mutated TR influences the arrangement of the α-helices

In the example *Test set 3: different TRs*, the binding of a mutated TR influences the arrangement of the α-helices, which became observable in the distribution parameter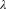 and the conical curvature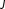. Especially by examining the conical curvature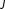, we noticed that the spherical curve of the MH2 in complex with the YAe62 has a higher conical curvature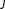. Simultaneously, the distribution parameter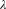 of the MH2 in complex with the YAe62 became earlier torsal than the MH2 in complex with B3K506. In our test set, the highest effect of a different TR binding to pMH is measured in the helix G-BETA. This is a consistent finding since the α-chain of the TR which adjacent to the α-helix G-BETA contains more mutations than the β-chain of the TR (17% vs. 7%, see Methods). This example illustrates that our method is suitable for the analysis of different TRs binding to the same MH.

### Quantification of severe helical disruptions

In the example *Test set 4: Helical disruption during a Molecular Dynamics simulation*, we investigated a severe helical disruption which occurred during an MD simulation. In this case, the disruption was easy to see even with the naked eye. However, our methodology allows scientists to quantify such severe deformations and make them directly comparable to other deformations.

### Which parameters are most appropriate for which kind of challenges?

For the analysis of a single part of a protein structure, we recommended to approximate it by a curve and calculate the curvature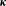 and the torsion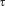. With these two measures, one is able to describe uniquely the underlying geometry.

For the analysis of the relative orientation between two structures, we recommend to approximate both structures by curves as in the single structure case but additionally span a ruled surface between them. On this basis, one can calculate the distribution parameter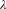 and the conical curvature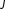, which describe uniquely the underlying geometry of the ruled surface.

In this study, we found only a minimal trend in the area between the α-helices; nevertheless, these measures may be meaningful for investigation of other complexes, where a contraction or relaxation between two protein structures occurs.

## Conclusion

Altogether our structural-based methodology offers a very detailed analysis of the alterations in α-helices for a broad variety of applications. By means of curvature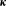 and torsion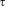, one is able to investigate the α-helices individually. To investigate the relative position of two α-helices, we provided the ruled surface together with four descriptive parameters (area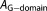, area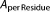, distribution parameter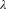, and conical curvature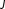). On the basis of these parameters, a deeper understanding of the interaction process between TR, peptide and MH is possible. The presented parameters will find a wide field of application, not only in classical TR/peptide/MH interactions, but also in challenges concerning superantigens, nonclassical MH functions, or even for helices not belonging to the MH superfamily.

## References

[1] Rudolph MG, Stanfield RL, Wilson IA (2006). Annu. Rev. Immunol.

[2] Germain RN (1994). Cell.

[3] Kaas Q, Ehrenmann F, Lefranc MP (2007). Brief. Funct. Genomic. Proteomic.

[4] Armstrong KM, Piepenbrink KH, Baker BM (2008). Biochem. J.

[5] Auphan-Anezin N, Mazza C, Guimezanes A, Barrett-Wilt GA, Montero-Julian F, Roussel A, Hunt DF, Malissen B, Schmitt-Verhulst AM (2006). Eur. J. Immunol.

[6] Knapp B, Omasits U, Schreiner W, Epstein MM (2010). PLoS One.

[7] Choudhuri K, van der Merwe PA (2007). Semin. Immunol.

[8] Pandini A, Fornili A, Kleinjung J (2010). BMC Bioinform.

[9] Pandini A, Fornili A, Fraternali F, Kleinjung J (2012). FASEB J.

[10] Kjer-Nielsen L, Clements CS, Brooks AG, Purcell AW, Fontes MR, McCluskey J, Rossjohn J (2002). J. Immunol.

[11] Knapp B, Omasits U, Bohle B, Maillere B, Ebner C, Schreiner W, Jahn-Schmid B (2009). Mol. Immunol.

[12] Knapp B, Fischer G, Van HD, Fae I, Maillere B, Ebner C, Schreiner W, Bohle B, Jahn-Schmid B (2012). BMC Immunol.

[13] Hischenhuber B, Frommlet F, Schreiner W, Knapp B (2012). Comput. Phys. Commun.

[14] Goldman BB, Wipke WT (2000). Proteins.

[15] Marathe A, Karandur D, Bansal M (2009). BMC Struct. Biol.

[16] Shazman S, Elber G, Mandel-Gutfreund Y (2011). Nucleic Acids Res.

[17] Schmidt NW, Tai KP, Kamdar K, Mishra A, Lai GH, Zhao K, Ouellette AJ, Wong GC (2012). J. Biol. Cxhem.

[18] Hausrath AC, Goriely A (2007). J. Struct. Biol.

[19] Lewiner T, Gomes J, Lopes H, Craizer M (2005). Comput. Graphics (Pergamon).

[20] Hurvich CM, Tsai CL (1989). Biometrika.

[21] Pottmann H, Wallner J (2001). Computational Line Geometry.

[22] Do Carmo MP (1976). Differential Geometry of Curves and Surfaces.

[23] Humphrey W, Dalke A, Schulten K (1996). J. Mol. Graph.

[24] Peternell M, Pottmann H, Ravani B (1999). CAD Comput. Aided Design.

[25] Chen HY, Pottmann H (1999). J. Comput. Appl. Math.

[26] Ranganathan S, Izotov D, Kraka E, Cremer D (2009). Proteins.

[27] Berman HM, Westbrook J, Feng Z, Gilliland G, Bhat TN, Weissig H, Shindyalov IN, Bourne PE (2000). Nucleic Acids Res.

[28] Ehrenmann F, Kaas Q, Lefranc MP (2010). Nucleic Acids Res.

[29] Velloso LM, Michaelsson J, Ljunggren HG, Schneider G, Achour A (2004). J. Immunol.

[30] Dai S, Huseby ES, Rubtsova K, Scott-Browne J, Crawford F, Macdonald WA, Marrack P, Kappler JW (2008). Immunity.

[31] Marrack P, Scott-Browne JP, Dai S, Gapin L, Kappler JW (2008). Annu. Rev. Immunol.

[32] Wilcox R (2012). Introduction to Robust Estimation & Hypothesis Testing.

[33] Vella JP, Spadafora-Ferreira M, Murphy B, Alexander SI, Harmon W, Carpenter CB, Sayegh MH (1997). Transplantation.

[34] Shoskes DA, Wood KJ (1994). Immunol. Today.

[35] Shirwan H, Leamer M, Wang HK, Makowka L, Cramer DV (1995). Transplantation.

[36] Opelz G, Wujciak T, Dohler B, Scherer S, Mytilineos J (1999). Rev. Immunogenet.

[37] Fangmann J, Dalchau R, Sawyer GJ, Priestley CA, Fabre JW (1992). Eur. J. Immunol.

[38] Rudolph MG, Wilson IA (2002). Curr. Opin. Immunol.

[39] Heemskerk MB, Roelen DL, Dankers MK, Claas JJ, van Rood, Doxiadis FH, Oudshoorn M (2005). Hum. Immunol.

[40] Elsner HA, DeLuca D, Strub J, Blasczyk R (2004). Bone Marrow Transplant.

[41] Varani L, Bankovich AJ, Liu CW, Colf LA, Jones LL, Kranz DM, Puglisi JD, Garcia KC (2007). Proc. Natl. Acad. Sci. USA.

[42] Thompson JA, Srivastava MK, Bosch JJ, Clements VK, Ksander BR, Ostrand-Rosenberg S (2008). Cancer Immunol. Immunother.

[43] Jardetzky TS, Brown JH, Gorga JC, Stern LJ, Urban RG, Chi YI, Stauffacher C, Strominger JL, Wiley DC (1994). Nature.

[44] Llewelyn M, Cohen J (2002). Lancet Infect. Dis.

[45] Russell JK, Pontzer CH, Johnson HM (1991). Proc. Natl. Acad. Sci. USA.

[46] Holoshitz DE, de Almeida J (2011). Self Nonself.

[47] Cardoso CS, de SM (2003). Tissue Antigens.

[48] Loconto J, Papes F, Chang E, Stowers L, Jones EP, Takada T, Kumanovics A, Fischer LK, Dulac C (2003). Cell.

[49] Simister NE, Jacobowitz IE, Ahouse JC, Story CM (1997). Biochem. Soc. Trans.

[50] Rudolph MG, Speir JA, Brunmark A, Mattsson N, Jackson MR, Peterson PA, Teyton L, Wilson IA (2001). Immunity.

[51] Painter CA, Stern LJ (2012). Immunol. Rev.

[52] Carven GJ, Stern LJ (2005). Biochemistry.

[53] Yaneva R, Springer S, Zacharias M (2009). Biopolymers.

[54] Adams EJ, Luoma AM (2013). Annu. Rev. Immunol.

[55] Quine JR (1999). J. Mol. Struct.: THEOCHEM.

[56] Quine JR, Cross TA, Chapman MS, Bertram R (2004). Bull. Math. Biol.

[57] Bates PW, Wei GW, Zhao S (2008). J. Comput. Chem.

[58] Koh E, Kim T (2005). Proteins.

[59] Koh E, Kim T, Cho HS (2006). Bioinformatics.

[60] Kjer-Nielsen L, Clements CS, Purcell AW, Brooks AG, Whisstock JC, Burrows SR, McCluskey J, Rossjohn J (2003). Immunity.

[61] Miller PJ, Pazy Y, Conti B, Riddle D, Appella E, Collins EJ (2007). J. Mol. Biol.

