# Supplementary material for: Differential Geometric Analysis of Alterations in MH α-Helices
Source: J Comput Chem. 2013 May 24;34(21):1862–79. doi: 10.1002/jcc.23328 (PMC3739936; doi:10.1002/jcc.23328)
Supplement: Supplementary file 1 [file jcc0034-1862-SD1.docx]

**Supplementary Material**

**for**

**Differential geometric analysis of alterations in MH α-helices**

B. Hischenhuber^1^, H. Havlicek^2^, J. Todoric^3,4^, S. Höllrigl-Binder^1,5^, W. Schreiner^1^, B. Knapp^1,6*^

^1^ Center for Medical Statistics; Informatics, and Intelligent Systems; Section for Biosimulation and Bioinformatics; Medical University of Vienna; Vienna; Austria

^2^ Faculty of Mathematics and Geoinformation; Institute of Discrete Mathematics and Geometry; Research Group for Differential Geometry and Geometric Structures; Vienna University of Technology; Vienna; Austria

^3^ Laboratory of Gene Regulation and Signal Transduction; Departments of Pharmacology and Pathology; School of Medicine; University of California; San Diego; USA

^4^ Department of Laboratory Medicine; Medical University Vienna; Vienna; Austria

^5^ Faculty of Mathematics and Geoinformation; Institute of Analysis and Scientific Computing; Research Group for Mathematical Modelling and Simulation; Vienna University of Technology; Vienna; Austria

^6^ Department of Statistics; Protein Informatics Group; University of Oxford; Oxford; United Kingdom

^*^corresponding email: bernhard.knapp@stats.ox.ac.uk

This Supplementary Material includes:

Figure S1: Representation of curvature $\kappa$ and torsion$\tau$ in different views

Figure S2**:** Area $A_{G-domain}$

Figure S3**:** Ruled surface

Figure S4: Director cone

Figure S5: Conical curvature $J$ zoomed in different ranges

Figure S6: Arc tangent representation of the conical curvature $J$

Figure S7: Results of *Test set 1*, MH1 class and weighted by b-factors

Figure S8: Results of *Test set 1,* MH2 class and weighted by b-factors

Figure S9: Visualization of the complex with the PDB-accession code 3c60 with two types of curves

Figure S10: RMSD (nm) of the complexes of Test set 1: How TRs deform MH α-helices

Figure S11**:** Results of *Test set 2: MH1 cross evaluation*

Figure S12**:** Results of *Test set 3: Different TR*

Figure S13**:** Results of *Test set 4: Helical disruption during a Molecular Dynamics simulation*

Figure S14: X-ray of vimentin coil 1A/1B fragment with a stabilizing mutation with the PDB-accession code 3s4r

Table S1**:** Retrospective calculation of the positions k on the striction curve of the ruled surface to the corresponding AA of the helix G-ALPHA1 and the helix G-ALPHA2 for MH1 complexes.

Table S2**:** Retrospective calculation of the positions k on the striction curve of the ruled surface to the corresponding AA of the helix G-ALPHA and the helix G-BETA for MH2 complexes.

Table S3: Resolution and b-factors of *Test case 2-4*

Table S4: RMSD (nm) of each complex of Test set 2 to the average structure


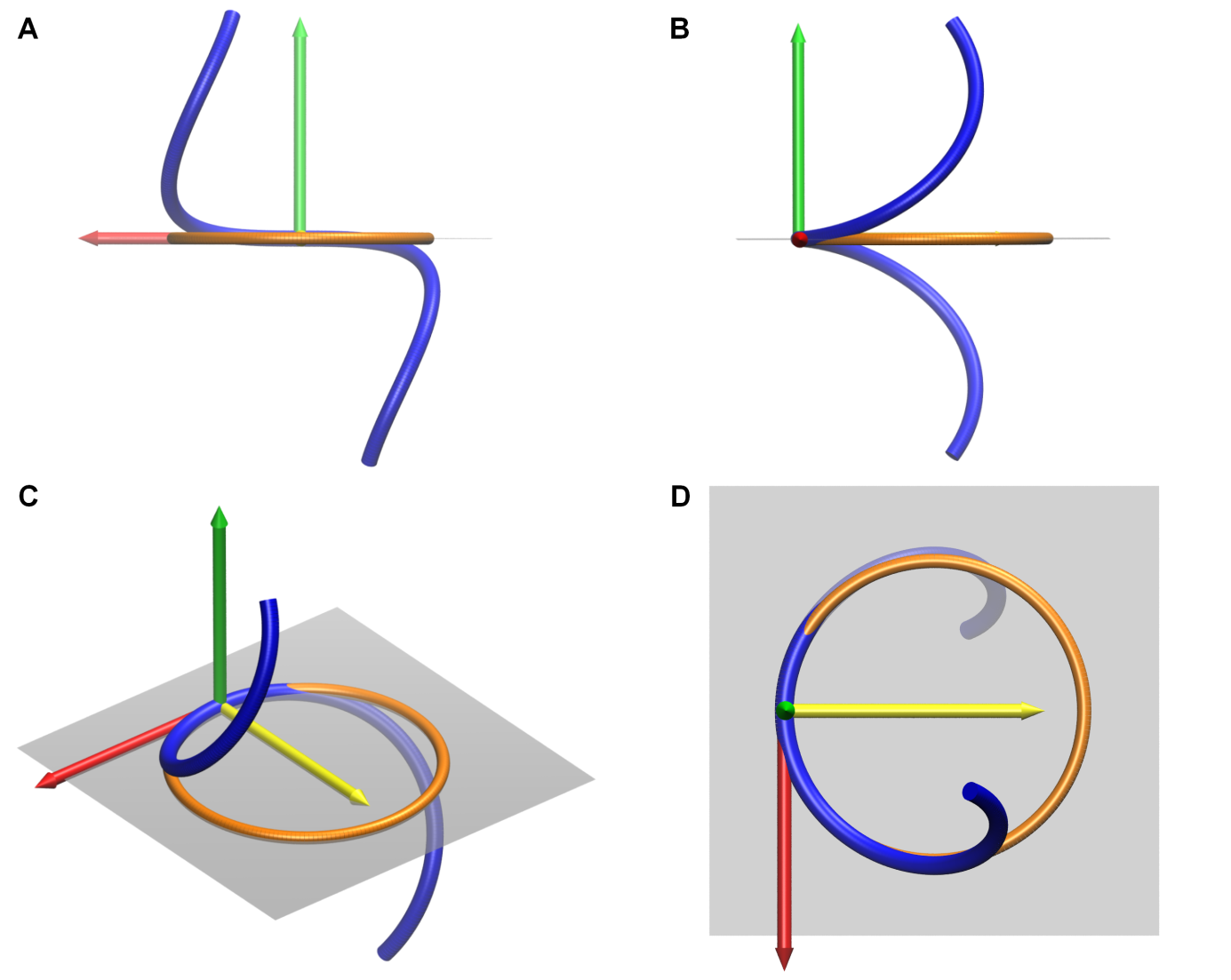


Figure S1. Representation of curvature $\boldsymbol{\kappa}$ and torsion$\boldsymbol{\tau}$ in different views: Curve (colored in blue) with a local coordinate system in a curve point spanned by tangent vector (colored in red), principal normal vector (colored in yellow) and binormal vector (colored in green). In the plane spanned by tangent vector and principal normal vector (colored in gray) the circle of curvature (colored in orange) is illustrated. (A) Front view. (B) Side view. (C) Inclined crack. (D) Top view.


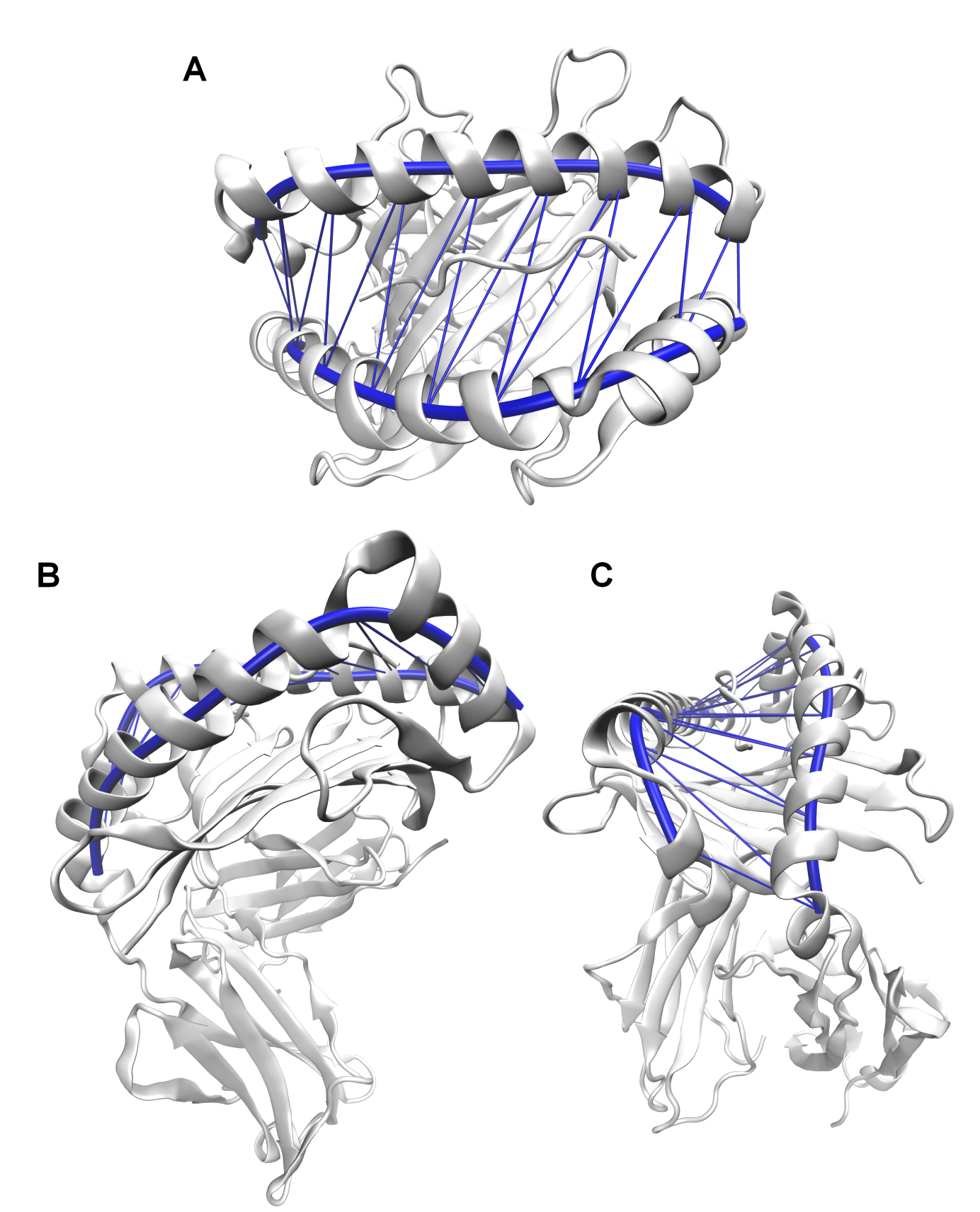


Figure S2. Area $\boldsymbol{A}_{\boldsymbol{G-domain}}$ illustrated in the crystal structure of the MH H-2Kb (colored in white). Two curves (colored in blue) representing the two α-helices of the MH. We calculated the area by a triangulation of the ruled surface between the two curves. (A) Top view. (B) Front view. (C) Side view.


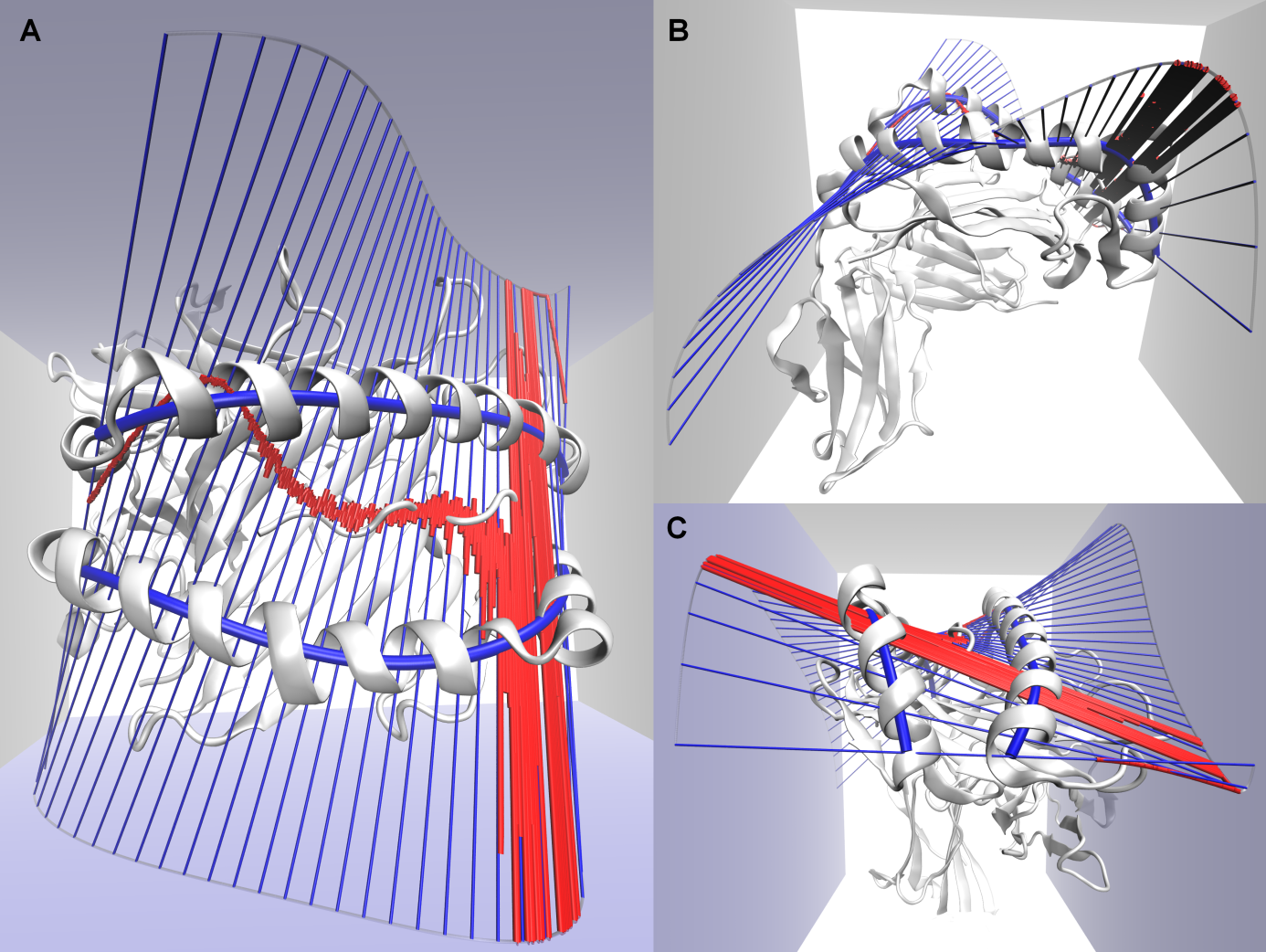


Figure S3. Ruled surface spanned by the curves (blue colored lines) representing the two α-helices of the MH H-2Kb (colored in white) with the PDB accession code 1s7q (compare *Test set 2: MH1 cross evaluation*). The rulings (coarse-grained illustrated in blue) originate from a movement of a straight line along the two curves. The striction curve (colored in red), representing the evolution of the distribution parameter $\boldsymbol{\lambda}$ and the conical curvature $\boldsymbol{J}$, illustrates in a graphical way the skew parts and the torsal parts (points of the striction curve converges to infinity). (A) Top view. (B) Front view. (C) Side view.


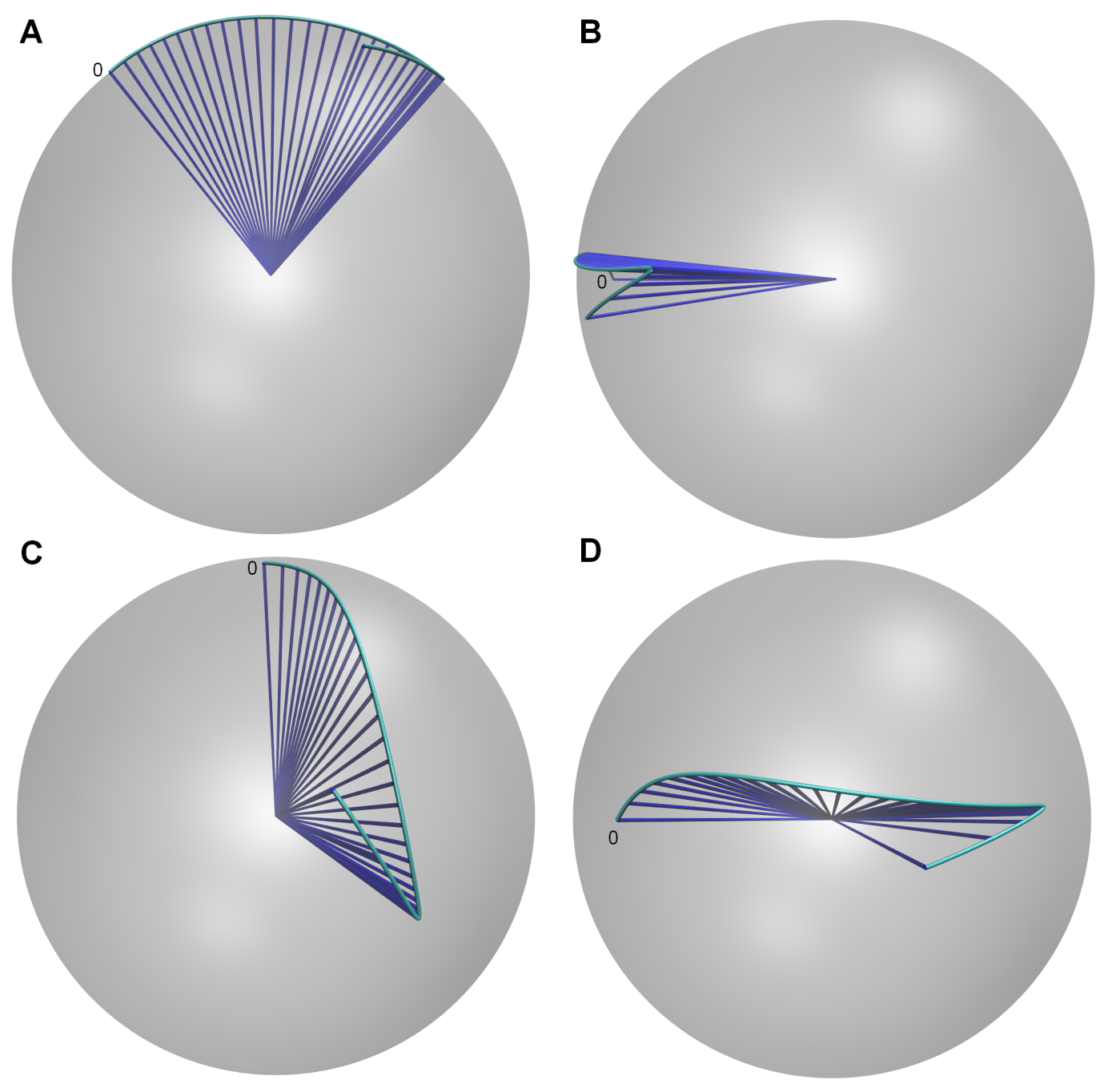


Figure S4. Director cone (course-grained blue rulings fixed in origin) with the spherical curve (cyan) on the unit sphere. The conical curvature measures the curvature on the unit sphere of the spherical curve. The zero marks the beginning of the ruled surface. (A) Front view. (B) Side view. (C) Inclined crack. (D) Top view.


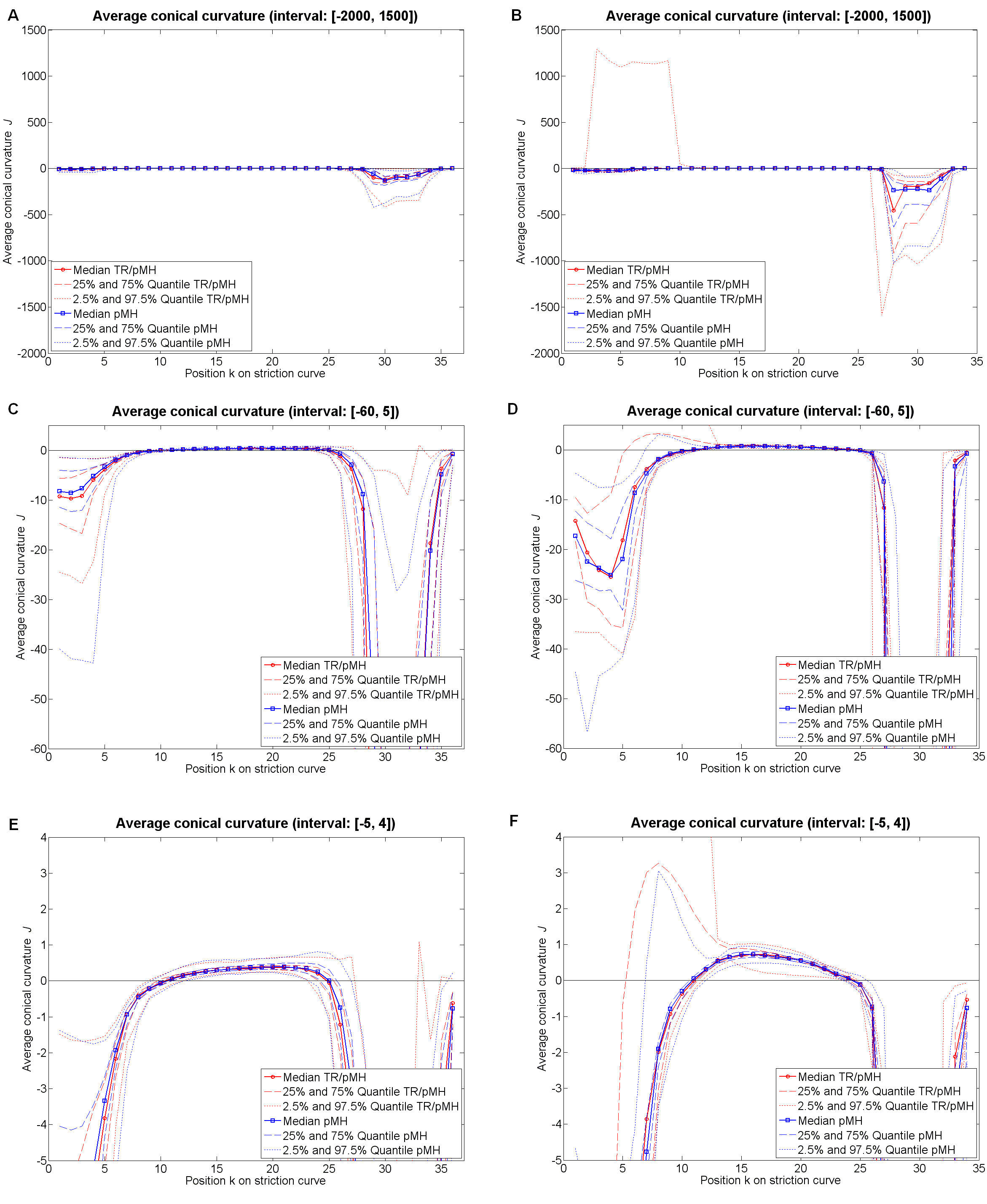


Figure S5. Conical curvature $\boldsymbol{J}$ (-) zoomed in different ranges. (A, C, E) Conical curvature $\boldsymbol{J}$ of MH1 class. (A) Zoom interval:
[-2000, 1500]. (C) Zoom interval: [-60, 5]. (E) Zoom interval: [-5, 4]. (B, D, F) Conical curvature $\boldsymbol{J}$ of MH2 class. (B) Zoom interval: [-2000, 1500]. (D) Zoom interval: [-60, 5]. (F) Zoom interval: [-5, 4].


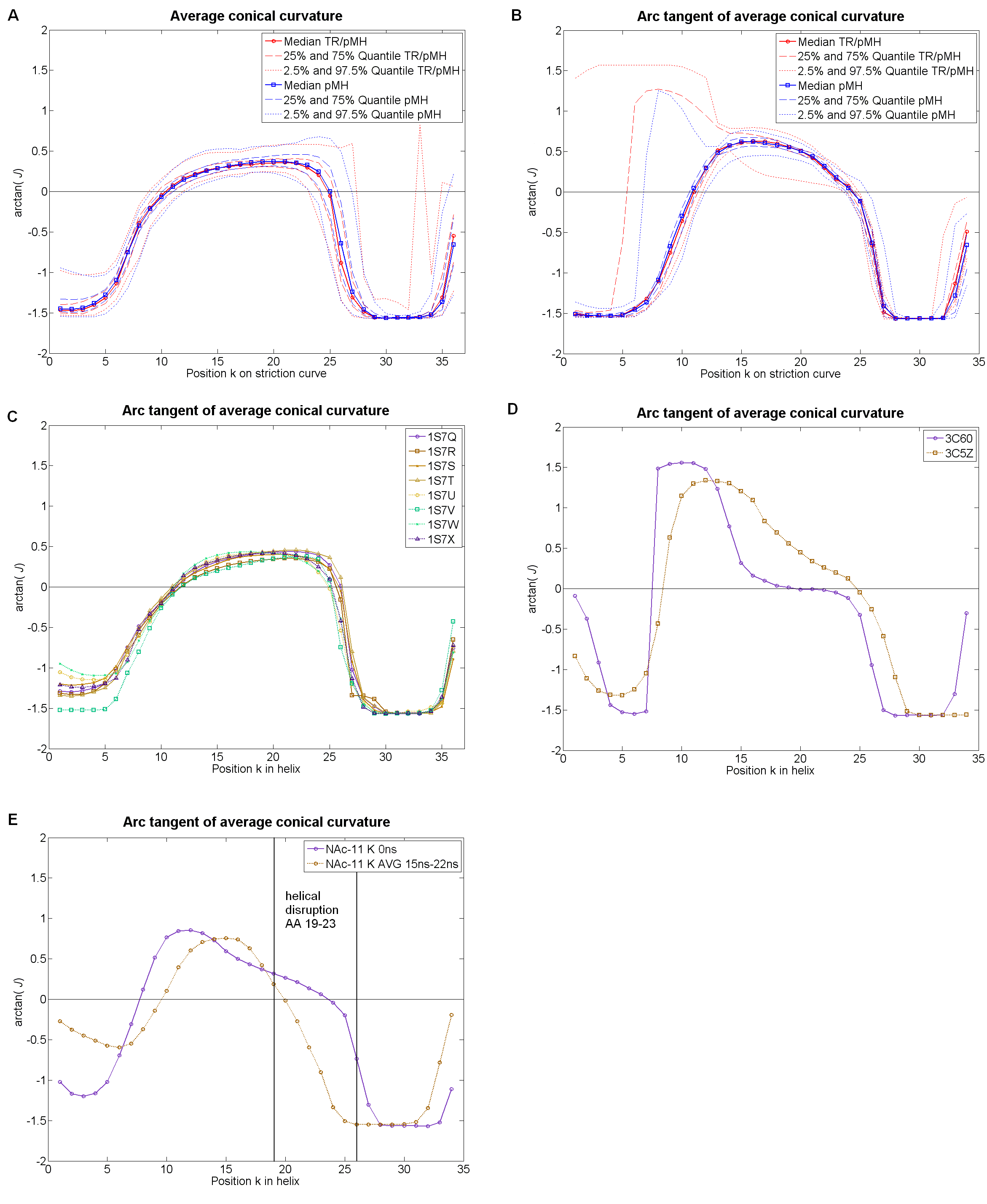


Figure S6. Arc tangent representation of the conical curvature $\boldsymbol{J}$ (-). (A) MH1 class. (B) MH2 class. (C) MH1 cross evaluation. (D) Different TRs (E) Helical disruption during a Molecular Dynamics simulation.


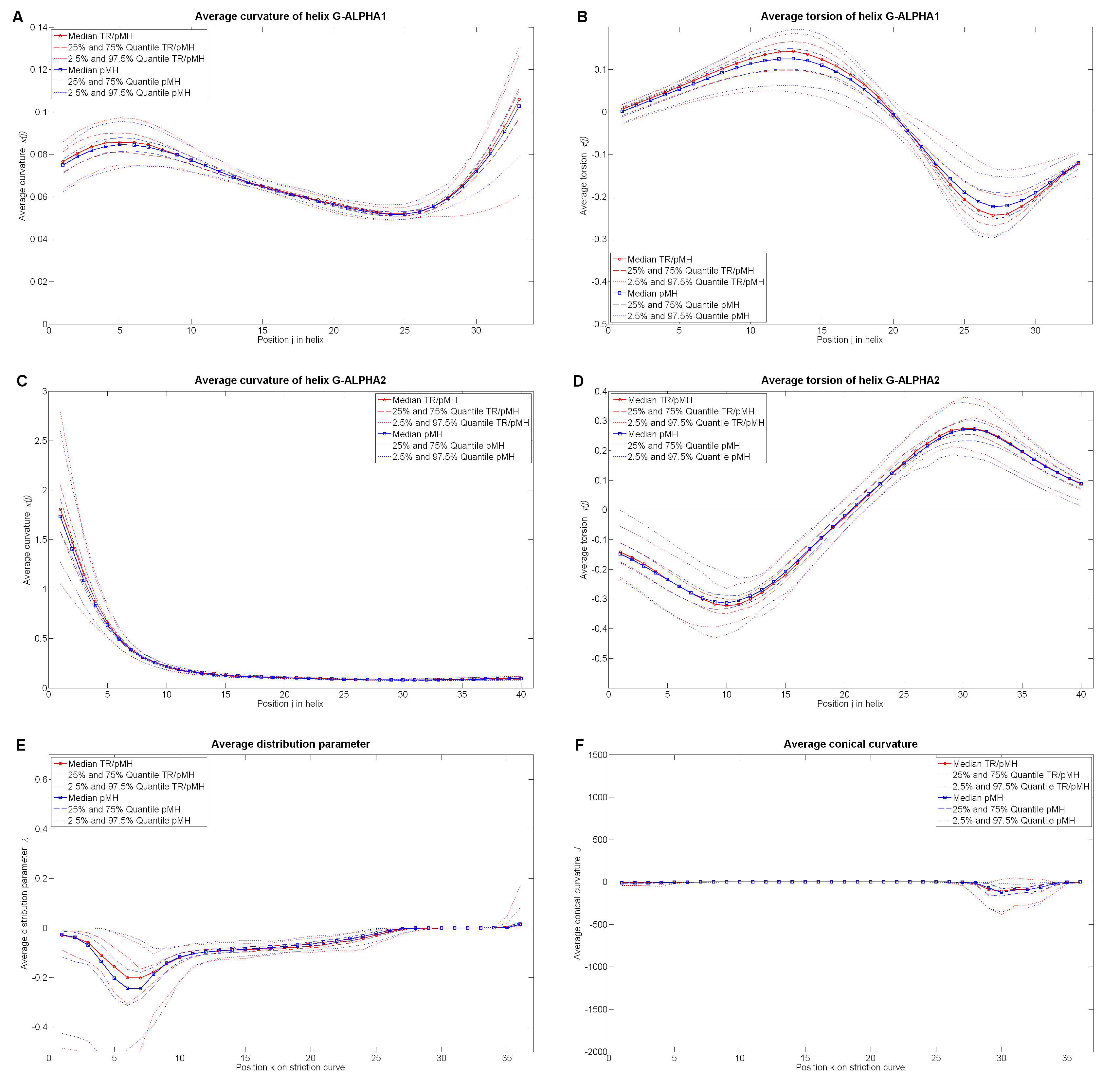


Figure S7. Results of Test set 1: How TRs deform MH α-helices, MH1 class and weighted by the inverse of the b-factors. We compared 321 pMH1 complexes (blue) against 52 TR/pMH1 complexes (red). The medians are depicted as solid lines the interquartile ranges are depicted as dashed lines and the IPR are depicted as dotted lines. The relative differences between the parameters remain almost similar (compare Figure 3). Often the information of the b-factors are incomplete, therefore we recommended in such cases the use of the standard curves without weights, as our results show. We provided an option in the software for both curve types.


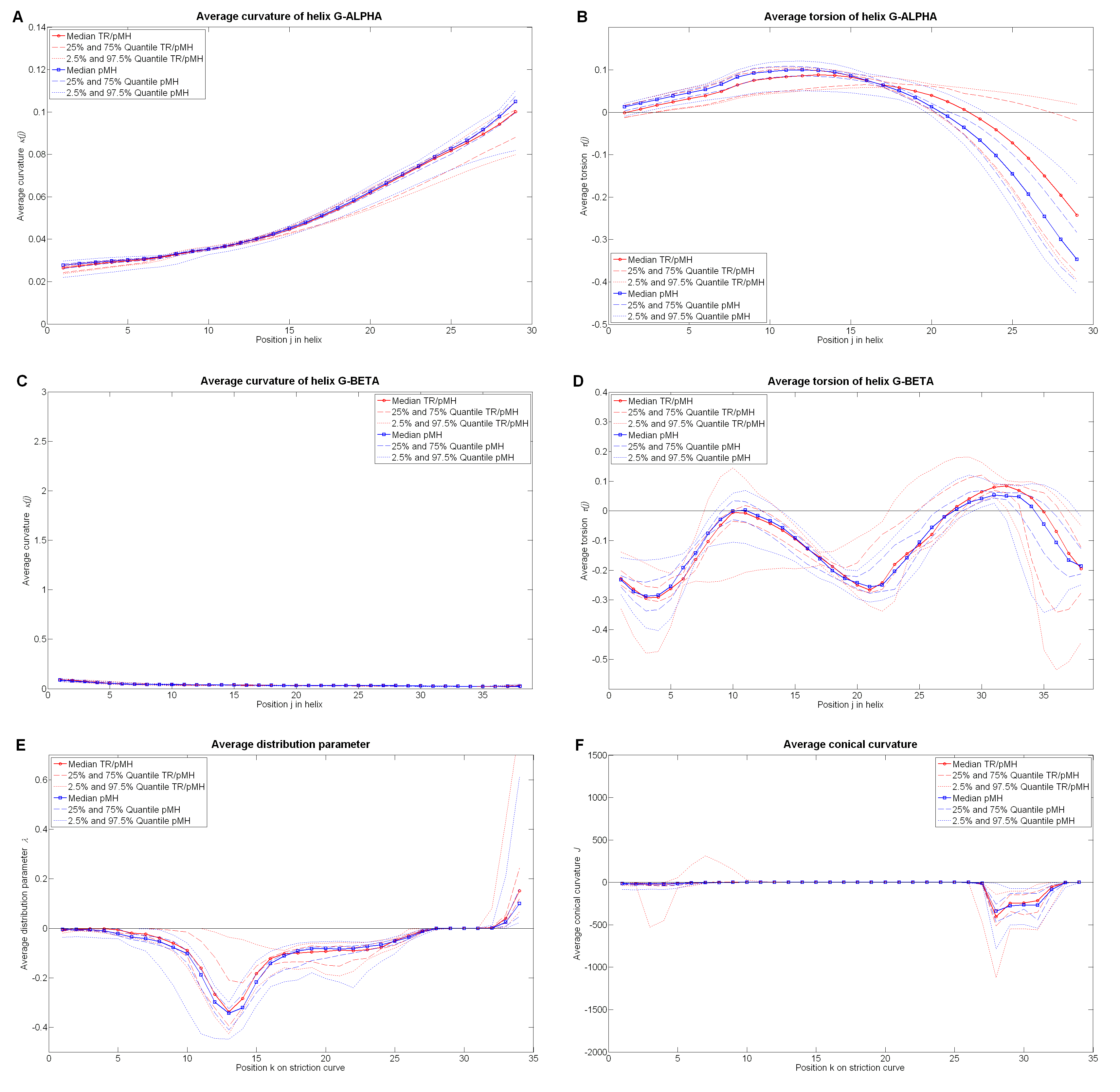


Figure S8. Results of Test set 1: How TRs deform MH α-helices, MH2 class and weighted by the inverse of the b-factors. We compared 18 pMH1 complexes (blue) against 10 TR/pMH1 complexes (red). The medians are depicted as solid lines the interquartile ranges are depicted as dashed lines and the IPR are depicted as dotted lines. The relative differences between the parameters remain almost similar (compare Figure 4). Often the information of the b-factors are incomplete, therefore we recommended in such cases the use of the standard curves without weights, as our results show. We provided an option in the software for both curve types.


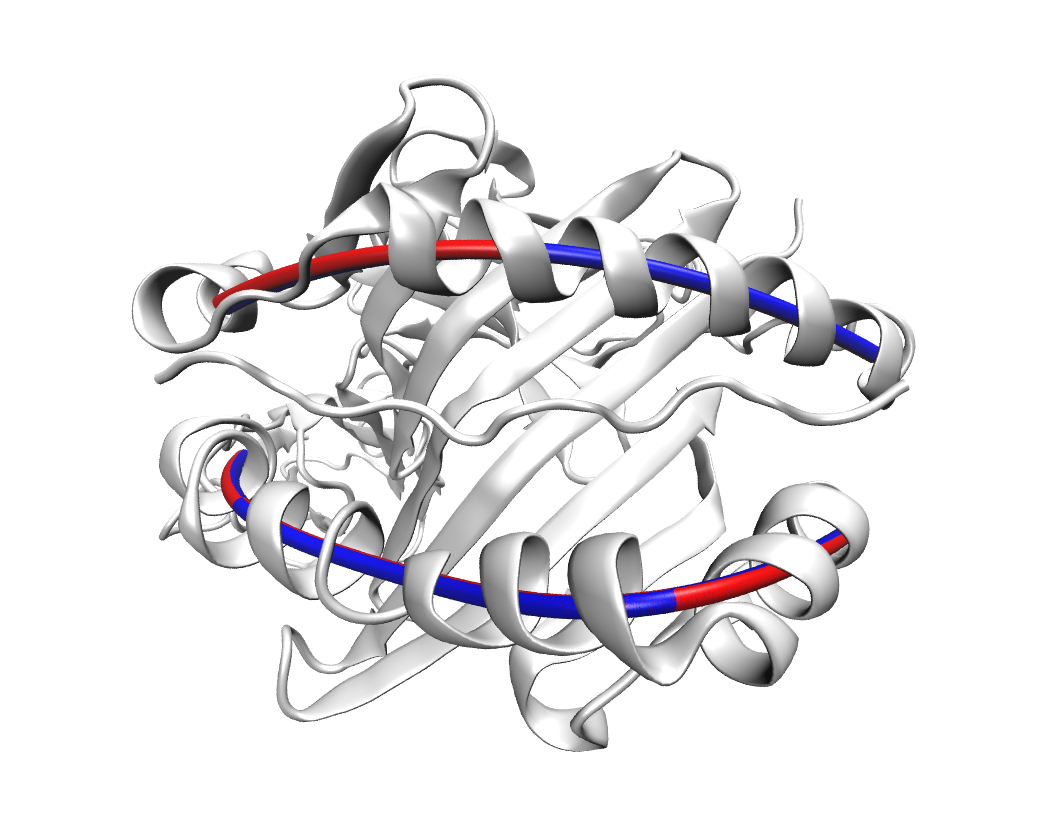


Figure S9. Visualization of the complex with the pdb-accession code 3c60 with two types of curves: We visualized the standard curves without weights in blue and the curves with the weighting of the inverse of the b-factor in red.


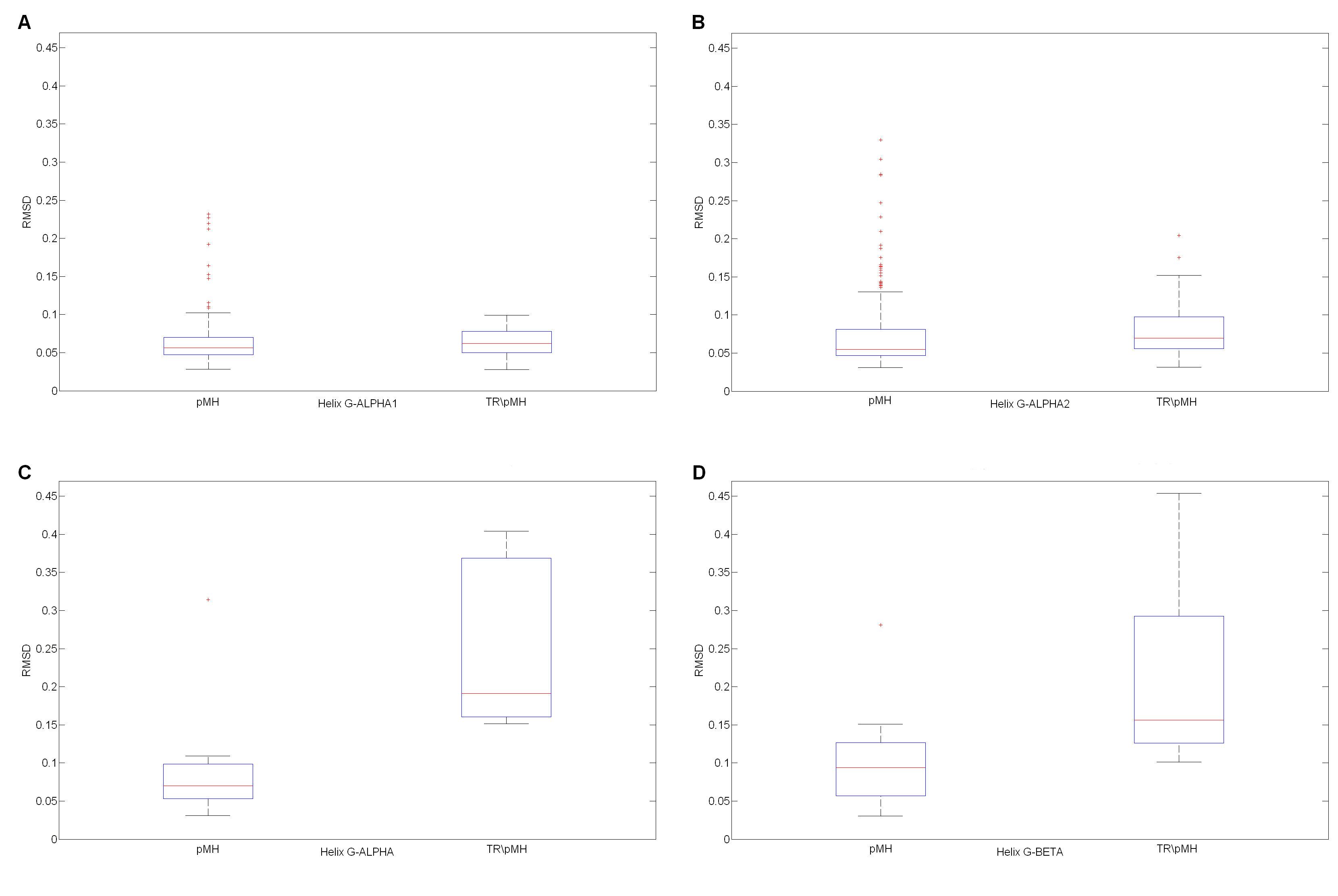


Figure S10. RMSD (nm) of the complexes of Test set 1: How TRs deform MH α-helices. We fitted all pMH1 complexes to the complex with the pdb-accession code 1a1m and all pMH2 complexes to the complex with the pdb-accession code 1a6a (in each case these complexes are the first one in alphabetical order of the PDB accession codes). Following we calculated the RMSD values to the average structure. (A) Comparison of the RMSD of the helix G-ALPHA1 between pMH1 and TR/pMH1 complexes. (B) Comparison of the RMSD of the helix G-ALPHA2 between pMH1 and TR/pMH1 complexes. (C) Comparison of the RMSD of the helix G-ALPHA between pMH2 and TR/pMH2 complexes. (D) Comparison of the RMSD of the helix G-BETA between pMH2 and TR/pMH2 complexes.


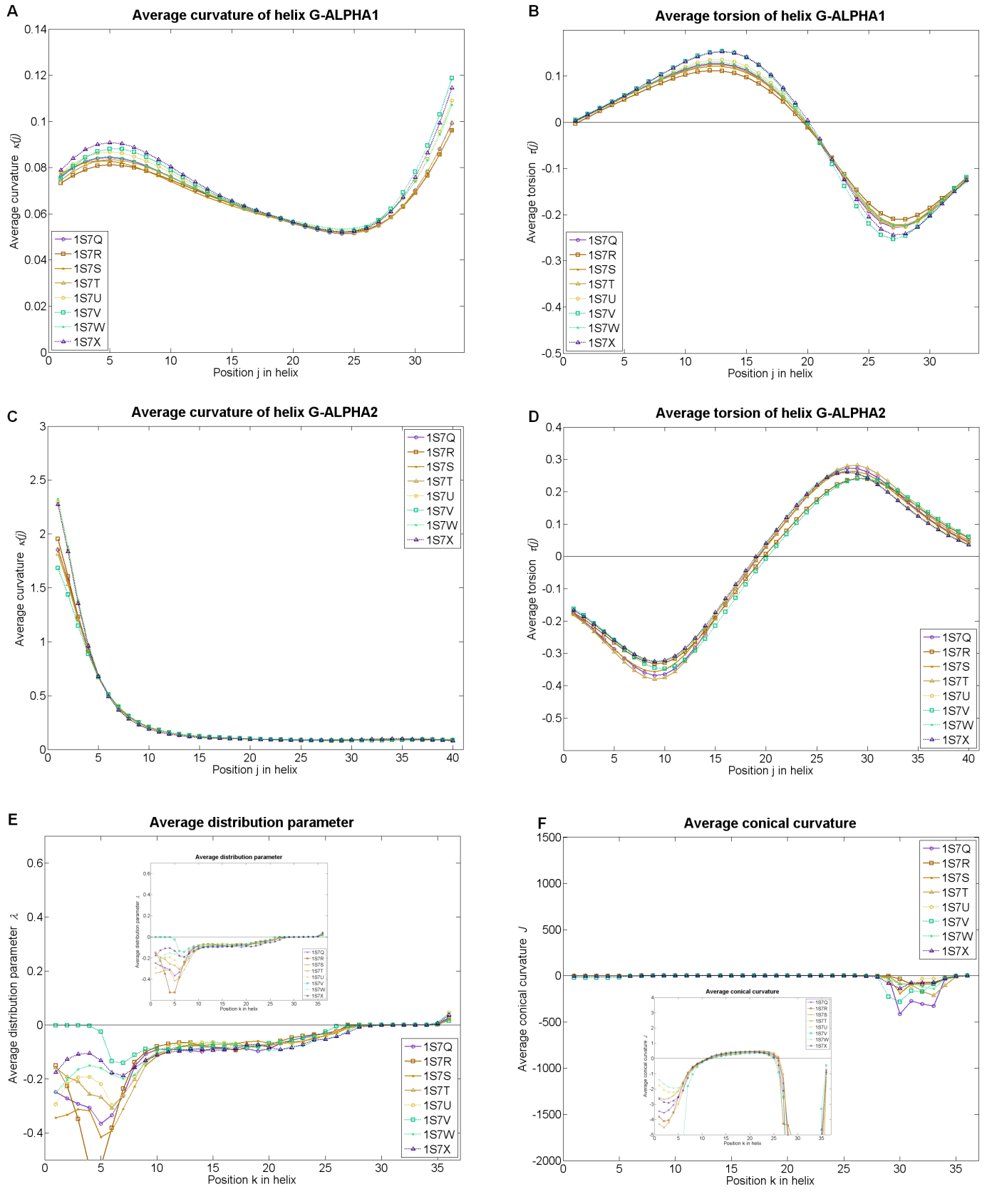


Figure S11. Results of Test set 2: MH1 cross evaluation. We compared eight pMH1 complexes (H-2Kb and H-2Db presenting each 4 different peptides). (A,B) Average curvature $\boldsymbol{\kappa}$ (nm^-1^) and average torsion$\boldsymbol{\tau}$ (nm^-1^) of helix G-ALPHA1 at 34 (j) positions obtained as moving average of the local parameters over a turn. (C,D) Average curvature $\boldsymbol{\kappa}$ (nm^-1^) and average torsion$\boldsymbol{\tau}$ (nm^-1^) of helix G-ALPHA2 at 40 (j) positions obtained as moving average of the local parameters over a turn. (E,F) Average distribution parameter $\boldsymbol{\lambda}$ (nm) and average conical curvature $\boldsymbol{J}$ (-) of the ruled surface at 36 (k) positions on the striction curve $\boldsymbol{c}_{\boldsymbol{*}}$ obtained as moving average of the local parameters over an average turn.


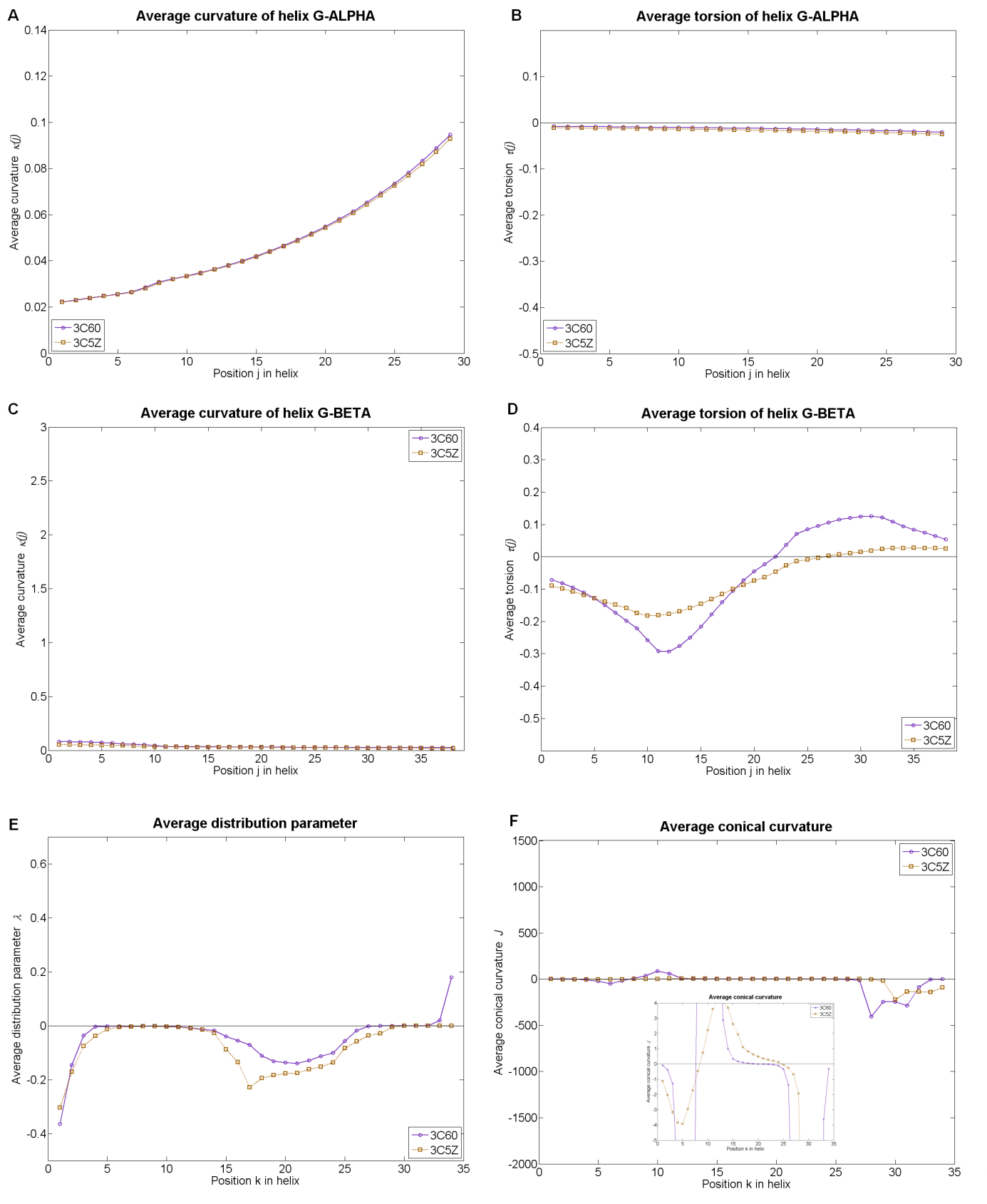


Figure S12. Results of Test set 3: Different TR. We compared two TR/pMH2 complexes (I-Ab presenting the peptide FEAQKAKANKAVD in complex with the TR YAe62 and in complex with the TR B3K506). (A,B) Average curvature $\boldsymbol{\kappa}$ (nm^-1^) and average torsion$\boldsymbol{\tau}$ (nm^-1^) of helix G-ALPHA at 29 (j) positions obtained as moving average of the local parameters over a turn. (C,D) Average curvature $\boldsymbol{\kappa}$ (nm^-1^) and average torsion$\boldsymbol{\tau}$ (nm^-1^) of helix G-BETA at 38 (j) obtained as moving average of the local parameters over a turn. (E,F) Average distribution parameter $\boldsymbol{\lambda}$ (nm) and total conical curvature $\boldsymbol{J}$ (-) of the ruled surface at 34 (k) positions on the striction curve $\boldsymbol{c}_{\boldsymbol{*}}$ originating by calculating the moving average of the local parameters over an average turn.


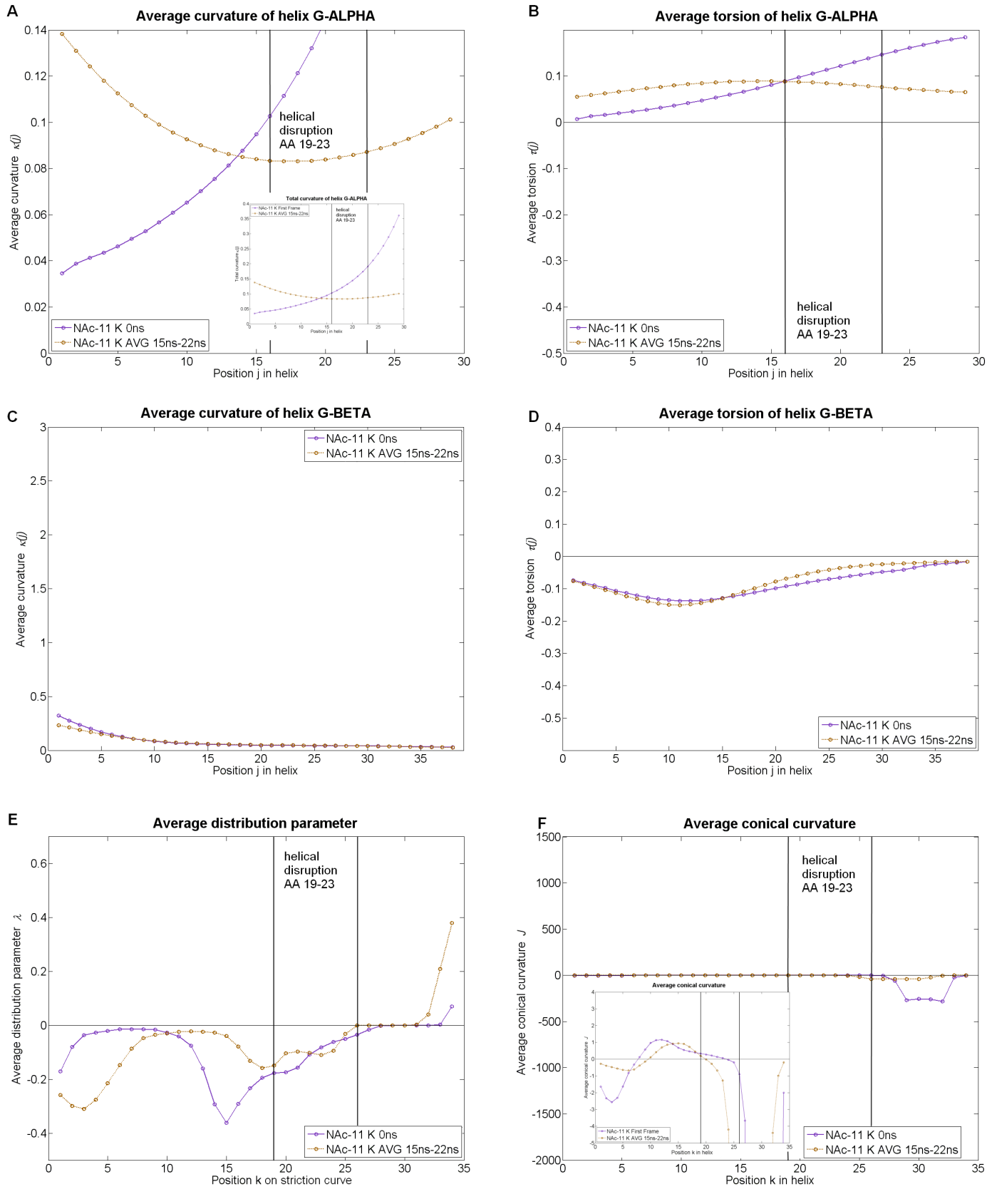


Figure S13. Results of Test set 4: Helical disruption during a Molecular Dynamics simulation. We compared two snapshots of the I-Au/MBP1-11 complex simulation (the snapshot at 0ns and an average snapshot of the time between the 15^th^ and the 22^nd^ ns). (A,B) Average curvature $\boldsymbol{\kappa}$ and average torsion$\boldsymbol{\tau}$ of helix G-ALPHA at 29 (j) positions obtained as moving average of the local parameters over a turn. (C,D) Average curvature $\boldsymbol{\kappa}$ and average torsion$\boldsymbol{\tau}$ of helix G-BETA at 38 (j) obtained as moving average of the local parameters over a turn. (E,F) Average distribution parameter $\boldsymbol{\lambda}$ and average conical curvature $\boldsymbol{J}$ of the ruled surface at 34 (k) positions on the striction curve $\boldsymbol{c}_{\boldsymbol{*}}$ obtained as moving average of the local parameters over an average turn.


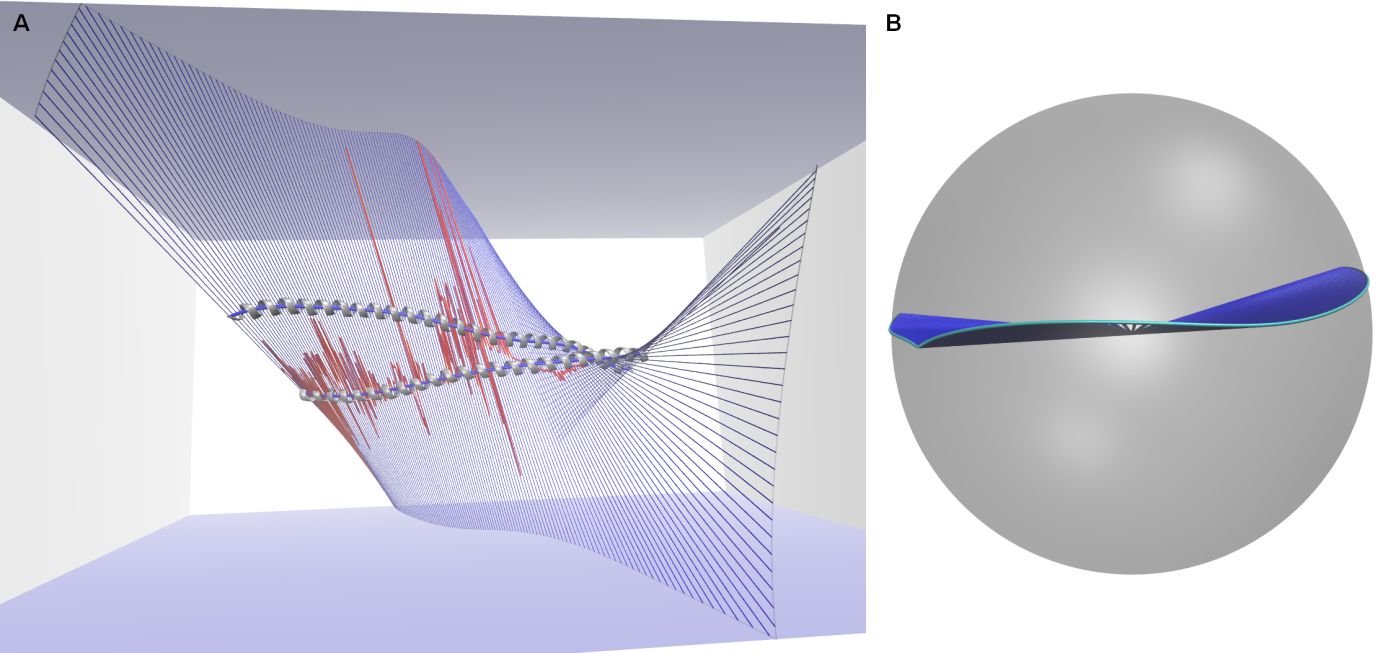


Figure S14. X-ray of vimentin coil1A/1B fragment with a stabilizing mutation with the PDB accession code 3s4r. (A) Ruled surface spanned by the curves (blue curves through the helices) with its striction curve (red). (B) Director cone (course-grained blue rulings fixed in origin) with the spherical curve (cyan) on the unit sphere.

Table S1. Retrospective calculation of the positions k on the striction curve $\boldsymbol{c}_{\boldsymbol{*}}$ of the ruled surface to the corresponding AA of the helix G-ALPHA1 and the helix G-ALPHA2 for MH1 complexes.

| **Position k on striction curve of ruled surface** | **AA of helix G-ALPHA1 consists of 36 AA** | **AA of helix G-ALPHA2 consists of 43 AA** |
| --- | --- | --- |
| 1 | 0.9-3.7 | 43-38.7 |
| 2 | 1.9-4.6 | 41.9-37.6 |
| 3 | 2.8-5.6 | 40.9-36.6 |
| 4 | 3.7-6.5 | 39.8-35.5 |
| 5 | 4.7-7.4 | 38.7-34.4 |
| 6 | 5.6-8.4 | 37.6-33.3 |
| 7 | 6.5-9.3 | 36.6-32.2 |
| 8 | 7.4-10.2 | 35.5-31.2 |
| 9 | 8.4-11.2 | 34.4-30.1 |
| 10 | 9.3-12.1 | 33.3-29.0 |
| 11 | 10.2-13.0 | 32.3-28.0 |
| 12 | 11.2-13.9 | 31.2-26.9 |
| 13 | 12.1-14.9 | 30.1-25.8 |
| 14 | 13.0-15.8 | 29.0-24.7 |
| 15 | 14.0-16.7 | 28.0-23.7 |
| 16 | 14.9-17.7 | 26.9-22.6 |
| 17 | 15.8-18.6 | 25.8-21.5 |
| 18 | 16.7-19.5 | 24.7-20.4 |
| 19 | 17.7-20.5 | 23.7-19.3 |
| 20 | 18.6-21.4 | 22.6-18.3 |
| 21 | 19.5-22.3 | 21.5-17.2 |
| 22 | 20.5-23.3 | 20.4-16.1 |
| 23 | 21.4-24.2 | 19.4-15.1 |
| 24 | 22.3-25.1 | 18.3-14.0 |
| 25 | 23.3-26.0 | 17.2-12.9 |
| 26 | 24.2-27.0 | 16.1-11.8 |
| 27 | 25.1-27.9 | 15.1-10.8 |
| 28 | 26.0-28.8 | 14.0-9.7 |
| 29 | 27.0-29.8 | 12.9-8.6 |
| 30 | 27.9-30.7 | 11.8-7.5 |
| 31 | 28.8-31.6 | 10.8-6.5 |
| 32 | 29.8-32.6 | 9.7-5.4 |
| 33 | 30.7-33.5 | 8.6-4.3 |
| 34 | 31.6-34.4 | 7.6-3.2 |
| 35 | 32.6-35.3 | 6.5-2.2 |
| 36 | 33.5-36 | 5.4-0 |

Table S2. Retrospective calculation of the positions k on the striction curve $\boldsymbol{c}_{\boldsymbol{*}}$ of the ruled surface to the corresponding AA of the helix G-ALPHA and the helix G-BETA for MH2 complexes.

| **Position k on striction curve of ruled surface** | **AA of helix G-ALPHA consists of 32 AA** | **AA of helix G-BETA consists of 41 AA** |
| --- | --- | --- |
| 1 | 0.9-3.6 | 41-36.4 |
| 2 | 1.8-4.4 | 39.9-35.3 |
| 3 | 2.7-5.3 | 38.7-34.2 |
| 4 | 3.6-6.2 | 37.6-33.0 |
| 5 | 4.4-7.1 | 36.4-31.9 |
| 6 | 5.3-8 | 35.3-30.8 |
| 7 | 6.2-8.9 | 34.2-29.6 |
| 8 | 7.1-9.8 | 33.0-28.5 |
| 9 | 8-10.7 | 31.9-27.3 |
| 10 | 8.9-11.6 | 30.8-26.2 |
| 11 | 9.8-12.4 | 29.6-25.1 |
| 12 | 10.7-13.3 | 28.5-23.9 |
| 13 | 11.6-14.2 | 27.3-22.8 |
| 14 | 12.4-15.1 | 26.2-21.6 |
| 15 | 13.3-16 | 25.1-20.5 |
| 16 | 14.2-16.9 | 23.9-19.4 |
| 17 | 15.1-17.8 | 22.8-18.2 |
| 18 | 16-18.7 | 21.6-17.1 |
| 19 | 16.9-19.6 | 20.5-15.9 |
| 20 | 17.8-20.4 | 19.4-14.8 |
| 21 | 18.7-21.3 | 18.2-13.7 |
| 22 | 19.6-22.2 | 17.1-12.5 |
| 23 | 20.4-23.1 | 15.9-11.4 |
| 24 | 21.3-24 | 14.8-10.3 |
| 25 | 22.2-24.9 | 13.7-9.1 |
| 26 | 23.1-25.8 | 12.5-8.0 |
| 27 | 24-26.7 | 11.4-6.8 |
| 28 | 24.9-27.6 | 10.3-5.7 |
| 29 | 25.8-28.4 | 9.1-4.6 |
| 30 | 26.7-29.3 | 8.0-3.4 |
| 31 | 27.6-30.2 | 6.8-2.3 |
| 32 | 28.4-31.1 | 5.7-1.1 |
| 33 | 29.3-31.9 | 4.6-0.1 |
| 34 | 30.2-32 | 3.4-0 |

Table S3. Resolution and b-factors of Test case 2-4

|  | Pdb accession code | Resolution of the complex (Å) | Average b-factors of helix G-ALPHA1/G-ALPHA (Å^2^) | Average b-factors of helix G-ALPHA2/G-BETA (Å^2^) |
| --- | --- | --- | --- | --- |
| Test case 2 | 1s7q | 1.99 | 14.65 | 14.22 |
|  | 1s7r | 2.95 | 22.35 | 24.67 |
|  | 1s7s | 1.99 | 20.11 | 20.28 |
|  | 1s7t | 2.30 | 24.44 | 25.72 |
|  | 1s7u | 2.20 | 20.33 | 26.81 |
|  | 1s7v | 2.20 | 19.72 | 22.08 |
|  | 1s7w | 2.40 | 13.25 | 23.04 |
|  | 1s7x | 2.41 | 26.16 | 34.39 |
| Test case 3 | 3c60 | 3.05 | 42.20 | 62.83 |
|  | 3c5z | 2.55 | 52.05 | 54.52 |
| Test case 4 | 1k2d | 2.20 | 50.47 | 61.25 |

Table S4. RMSD (nm) of each complex of Test set 2 to the average structure

|  | 1s7q | 1s7r | 1s7s | 1s7t | 1s7u | 1s7v | 1s7w | 1s7x |
| --- | --- | --- | --- | --- | --- | --- | --- | --- |
| RMSD of helix G-ALPHA1 | 0.0556 | 0.0473 | 0.0592 | 0.0437 | 0.0625 | 0.0417 | 0.0612 | 0.0713 |
| RMSD of helix G-ALPHA2 | 0.0454 | 0.0459 | 0.0421 | 0.0450 | 0.0589 | 0.0621 | 0.0660 | 0.0653 |
